# Engineering Photothermal Catalytic CO_2_ Nanoreactor for Osteomyelitis Treatment by In Situ CO Generation

**DOI:** 10.1002/advs.202402256

**Published:** 2024-04-22

**Authors:** Fan Zhuang, Luxia Jing, Huijing Xiang, Cuixian Li, Beilei Lu, Lixia Yan, Jingjing Wang, Yu Chen, Beijian Huang

**Affiliations:** ^1^ Department of Ultrasound Zhongshan Hospital Fudan University and Shanghai Institute of Medical Imaging Shanghai 200032 P. R. China; ^2^ Materdicine Lab School of Life Sciences Shanghai University Shanghai 200444 P. R. China; ^3^ Oujiang Laboratory (Zhejiang Lab for Regenerative Medicine Vision and Brain Health) Wenzhou Institute of Shanghai University Wenzhou Zhejiang 325088 P. R. China; ^4^ Shanghai Institute of Materdicine Shanghai 200051 P. R. China

**Keywords:** anti‐inflammation, bacterial inhibition, carbon monoxide generation, MXenes, photothermal CO_2_ catalysis

## Abstract

Photocatalytic carbon dioxide (CO_2_) reduction is an effective method for in vivo carbon monoxide (CO) generation for antibacterial use. However, the available strategies mainly focus on utilizing visible‐light‐responsive photocatalysts to achieve CO generation. The limited penetration capability of visible light hinders CO generation in deep‐seated tissues. Herein, a photothermal CO_2_ catalyst (abbreviated as NNBCs) to achieve an efficient hyperthermic effect and in situ CO generation is rationally developed, to simultaneously suppress bacterial proliferation and relieve inflammatory responses. The NNBCs are modified with a special polyethylene glycol and further embellished by bicarbonate (BC) decoration via ferric ion‐mediated coordination. Upon exposure to 1064 nm laser irradiation, the NNBCs facilitated efficient photothermal conversion and in situ CO generation through photothermal CO_2_ catalysis. Specifically, the photothermal effect accelerated the decomposition of BC to produce CO_2_ for photothermal catalytic CO production. Benefiting from the hyperthermic effect and in situ CO production, in vivo assessments using an osteomyelitis model confirmed that NNBCs can simultaneously inhibit bacterial proliferation and attenuate the photothermal effect‐associated pro‐inflammatory response. This study represents the first attempt to develop high‐performance photothermal CO_2_ nanocatalysts to achieve in situ CO generation for the concurrent inhibition of bacterial growth and attenuation of inflammatory responses.

## Graphical abstract

The distinct photothermal CO_2_ nanocatalysts, NNBCs, is engineered to generate hyperthermia and in situ CO, tackling bacterial proliferation and inflammation simultaneously. Under 1064 nm laser irradiation, these NNBCs produce heat that facilitates bicarbonate decomposition to CO_2_. Evidence from the osteomyelitis models underscore their ability to stifle bacterial growth and soothe inflammation, marking a pioneering approach in antibacterial and anti‐inflammatory nanomedicine.

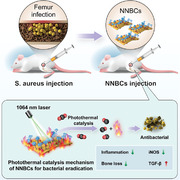

## 1 Introduction

Carbon monoxide (CO), one of the deadliest gases, which is toxic to humans at high doses, is increasingly being acknowledged as a crucial signaling molecule, akin to nitric oxide (NO) and hydrogen sulfide (H_2_S), with essential physiological roles.^[^ ^1^ ^]^ In addition, CO has been explored as an effective therapeutic agent because of its cytoprotective, anti‐inflammatory, anticancer, and antibacterial properties.^[^ ^2^ ^]^ As early clinical trials of CO administration through inhalers faced challenges in transporting accurate amounts of CO to patients, safer CO delivery alternatives are needed.^[^ ^3^ ^]^ Photocatalytic and electrocatalytic carbon dioxide (CO_2_) reduction has proven promising for in vivo CO production.^[^ ^4^ ^]^ By varying external light exposure, the generation of CO can be precisely regulated, making this an attractive strategy for in situ CO generation.^[^ ^5^ ^]^ Nevertheless, current investigations mainly focus on developing visible‐light‐responsive photocatalysts to achieve efficient conversion of CO_2_ to CO in vivo. The limited penetration capability of visible light severely hinders in situ CO generation in deep‐seated tissues.^[^ ^6^ ^]^ Therefore, it is vital to develop alternative CO‐releasing nanosystems that can be catalyzed by a near‐infrared (NIR) laser for in situ CO production in deep‐seated tissues.

Photothermal catalysis is an efficient alternative to traditional thermocatalysis and photocatalysis in energy conversion owing to its superior throughput, unprecedented light‐harvesting efficiency, and moderate reaction conditions.^[^ ^7^ ^]^ The efficiency of a photothermal catalyst depends largely on its photothermal and intrinsic thermocatalytic performance. Substantial endeavors have been devoted to the development of photothermal CO_2_ catalysts.^[^ ^8^ ^]^ To date, most developed photothermal catalysts involve a variety of transition metal oxide nanoparticles, plasmonic metal nanoparticles, and organic conjugated polymers.^[^ ^9^ ^]^ Although some successful paradigms have been realized for metal nanoparticle trapped metal‐organic frameworks, key bottlenecks remain to be addressed.^[^ ^10^ ^]^ To achieve an effective catalytic reaction, direct integration of the catalytic and photothermal centers is necessary. Currently, there are few efficient catalysts with photothermal properties, and these photothermal nanomaterials are frequently unable to effectively perform CO_2_ catalysis. Therefore, it is highly desirable to rationally design photothermal CO_2_ catalysts that combine photothermal conversion and catalytic activity for in situ CO generation under NIR laser irradiation.

MXenes, including early transition‐metal carbides, nitrides, and carbonitrides with planar structures, have garnered significant interest because of their chemical diversity, excellent photothermal conversion properties, and high biocompatibility.^[^ ^11^ ^]^ Among these, niobium carbide (Nb_2_C) nanosheets stand out for their excellent photothermal conversion performance, making them desirable candidates that can serve as safe antibacterial biomaterials for effectively bacterial elimination and wound healing.^[^ ^12^ ^]^ The ultrathin morphology of Nb_2_C has the potential do decrease carrier diffusion length and reduce electron‐hole recombination rates, while also facilitating atomic‐level element doping to effectively modulate CO_2_ reduction activity and selectivity. Additionally, presence of metal nanoparticles on the MXene surface can enhance photocatalytic reactions through the potential transfer of hot charge carriers, excited by localized surface plasmon resonance (LSPR), from the MXenes to the metal nanoparticles. The enhancement of photocatalytic reactions can be achieved by depositing metal nanoparticles onto MXenes, facilitating the transfer of LSPR‐excited hot charge carriers and reactants from MXenes to the metal nanoparticles.^[^ ^13^ ^]^ However, the use of MXenes as a platform for metal nanoparticles to achieve efficient photothermal CO_2_ catalysis has not been achieved.

**Scheme 1:**
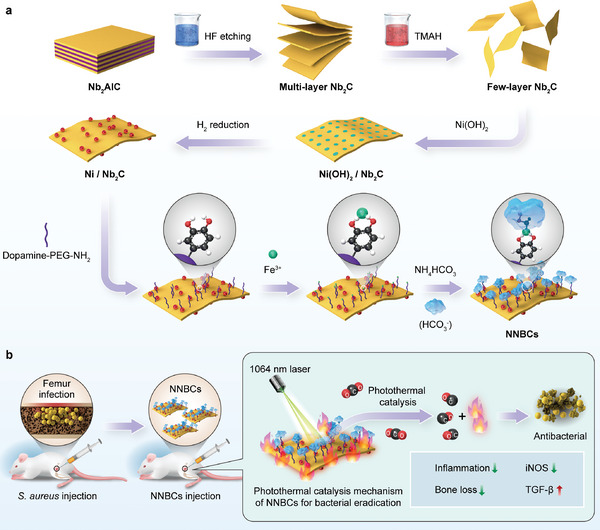
Schematic illustration of photothermal CO_2_ catalytic nanoreactor NNBCs for osteomyelitis treatment. a) Synthetic procedures of NNBCs, and b) the therapeutic mechanism of NNBCs under 1064 nm laser irradiation.

In this study, a photothermal CO_2_ catalytic nanoplatform (denoted as NNBCs) was intelligently engineered to realize photothermal effect‐augmented and CO‐mediated antibacterial therapies. Nb_2_C MXene nanosheet‐supported nickel (Ni) nanoparticles were decorated with a special polyethylene glycol (NH_2_‐PEG‐DPA) and then modified with bicarbonate (BC) through ferric ion (Fe^3+^)‐mediated coordination to fabricate NNBCs (**Scheme** **1**a). Under 1064 nm laser exposure, the Nb_2_C nanosheet‐supported Ni nanoparticles in NNBCs facilitated in situ CO generation via efficient photothermal CO_2_ catalysis. Specifically, the hyperthermia effect promoted the decomposition of BC to self‐supply CO_2_ in the vicinity of the nanoplatform, thus boosting photothermal catalytic CO generation. Profiting from efficient photothermal conversion and in situ CO generation under single laser exposure to NIR light in the second biological window (NIR‐II), NNBCs can simultaneously achieve thermal destruction of bacteria and attenuation of hyperthermia effect‐induced inflammation (Scheme 1b). This work connects photothermal‐conversion nanoplatforms with photothermal CO_2_ catalysis to improve high‐efficiency catalytic reactions to concurrently achieve enhanced antibacterial efficacy and alleviate the inflammatory response.

## 2 Results and Discussion

**Figure 1:**
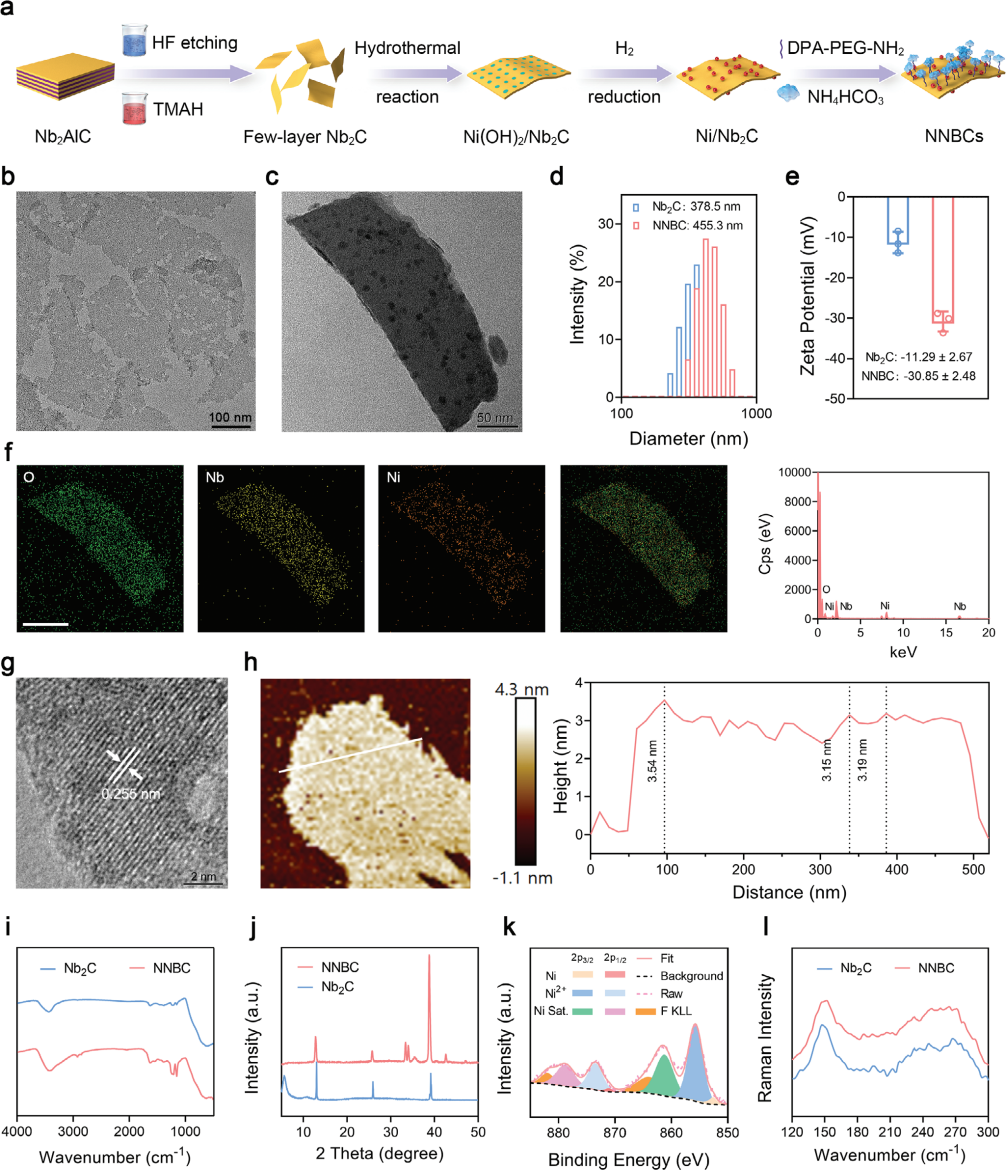
Characterization of NNBCs. a) Fabrication route of NNBCs. b,c) Transmission electron microscopy (TEM) image of few‐layer b) Nb_2_C and c) NNBCs. d) Size distribution of few‐layer Nb_2_C and NNBCs. e) Zeta potential of few‐layer Nb_2_C and NNBCs (n = 3). f) Elemental mapping images and the corresponding energy dispersive spectrum of NNBCs. g) High‐resolution TEM image of NNBCs. h) Atomic force microscopy (AFM) image and the corresponding thickness distribution of NNBCs. i) Fourier transform infrared (FTIR) spectra, and j) X‐ray diffraction (XRD) patterns of few‐layer Nb_2_C and NNBCs. k) High‐resolution X‐ray photoelectron spectroscopy (XPS) of Ni element for NNBCs. l) Raman spectra of few‐layer Nb_2_C and NNBCs.

As depicted in **Figure** **1**a, few‐layer Nb_2_C was obtained using a modified chemical exfoliation strategy, which involved the removal of Al layers in a MAX‐phase Nb_2_AlC precursor via hydrofluoric acid (HF) etching, followed by intercalation in tetramethylammonium hydroxide (TMAH).^[^ ^14^ ^]^ To fabricate Ni/Nb_2_C, Ni(NO_3_)_2_•6H_2_O, ammonium hydroxide solution (NH_3_•H_2_O), polyvinylpyrrolidone (PVP), and few‐layer Nb_2_C were hydrothermally reacted. The obtained Ni(OH)_2_/Nb_2_C samples were reduced at 300 °C for 3 h, under a H_2_ atmosphere to acquire Ni/Nb_2_C.^[^ ^15^ ^]^ Subsequently, NH_2_‐PEG‐DPA was grafted onto the prepared Ni/Nb_2_C via electrostatic interactions (Figure S1, Supporting Information). The successful modification of BC on the surface of Ni/Nb_2_C was achieved to obtain biofunctional nanosystems (denoted as NNBCs) by the introducing Fe^3+^ as the coordination center of dopamie (DPA) and BC.^[^ ^16^ ^]^ After the successful establishment of NNBCs, the size and morphology of the few‐layer Nb_2_C and NNBCs were observed by transmission electron microscopy (TEM). TEM images revealed the typical planar topology of the Nb_2_C and NNBCs, each measuring ≈378 and 450 nm on average (Figure 1b,c). Subsequently, the hydrodynamic diameter distribution and ζ potential of the few‐layer Nb_2_C and NNBCs were investigated. The incorporation of Ni nanoparticles and BC resulted in a slight increase in the hydrodynamic diameter and a minor fluctuation in the ζ potential of the NNBCs (Figure 1d,e). Moreover, elemental mapping images revealed the homogeneous dispersion of O, Nb, and Ni elements in the planar topology of the NNBCs (Figure 1f). High‐resolution TEM (HRTEM) images reveal that the spacing between two neighboring planes of Ni nanoparticles in a specific orientation measures 0.255 nm, aligning with the lattice distance of the Ni (111) plane (Figure 1g).^[^ ^17^ ^]^

As shown in Figure 1h, the thickness of the NNBCs was determined to be 3.2 nm using atomic force microscopy (AFM). The successful fabrication of the NNBCs was further confirmed by Fourier transform infrared (FTIR) spectroscopy (Figure 1i). Additionally, the successful loading of Ni was further demonstrated by the X‐ray diffraction (XRD) patterns of the few‐layer Nb_2_C and NNBCs (Figure 1j). Based on the energy position and peak pattern characteristics of the Ni_2p3/2_ spectrum, Ni was primarily composed of metallic Ni and Ni (OH)_2_ (Figure 1k). In addition, Raman spectroscopy of Nb_2_C and NNBCs presented the typical ω1 and ω4 vibration patterns, illustrating the stable structure of NNBCs (Figure 1l). Furthermore, the stability of the NNBCs in various incubation solutions was assessed by dynamic light scattering analysis. The hydrodynamic size of the NNBCs exhibited consistent stability over seven days without any notable variations, indicating the remarkable stability of the NNBCs under various physiological conditions (Figure S2, Supporting Information).

**Figure 2:**
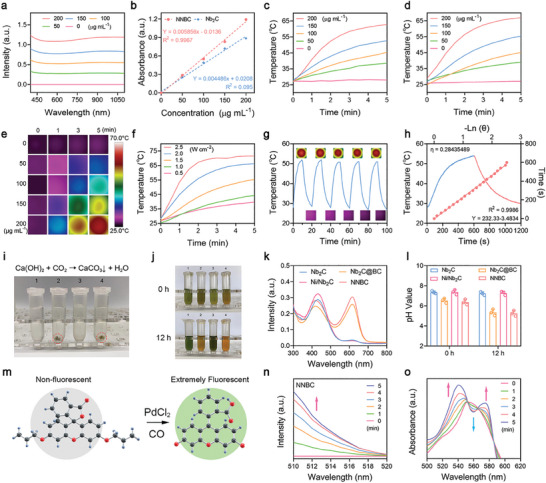
Photothermal‐conversion and CO generation capability of NNBCs. a) Ultraviolet‐visible (UV–vis)‐NIR absorption spectra of NNBCs at various concentrations. b) Molar absorption coefficients of few‐layer Nb_2_C and NNBCs. c,d) Photothermal curves of c) few‐layer Nb_2_Cs and d) NNBCs under 1064 nm laser irradiation. e) The thermal images of different concentrations of NNBCs under 1064 nm laser irradiation. f) Photothermal curves of NNBCs under irradiation by a NIR laser at diverse power densities. g) Heating curve of 5 on/off cycles of NNBCs under 1064 nm laser irradiation. h) Photothermal‐conversion curve of NNBCs under 1064 nm laser exposure and the corresponding linear relationship between the cooling duration and logarithm of the temperature change. i) Photographs of various mixtures after 1064 nm laser illumination and addition of Ca (OH)_2_. j) Photographs of various mixtures after 1064 nm laser irradiation and addition of BTB solution for 0 h (up) and 12 h (down). k) The corresponding UV–vis absorption spectra of i) (1: Nb_2_C; 2: Nb_2_C@BC; 3: Ni/Nb_2_C; 4: NNBCs). l) pH levels of the supernatant after various treatments for 0 and 12 h (n = 3). Data are presented as mean ± SD. m) Schematic illustration of fluorescent detection of CO with a CO probe. n) Fluorescent spectra changes of NNBCs together with CO probe and PdCl_2_ under 1064 nm laser irradiation. o) CO release from NNBCs as measured by myoglobin assay.

Due to the broad and robust absorption capacity of NNBCs in the NIR region, we recorded the temperature variation of NNBCs under 1064 nm laser irradiation to assess their photothermal conversion capability (**Figure** **2**a,b). Upon exposed to the 1064 nm laser illumination for 5 min, the temperature of NNBCs increased from 28.9 to 67 °C at a concentration of 200 µg mL^−1^ (Figure 2d,e). Subsequently, we monitored and recorded the temperature variations at different concentrations of Nb_2_C and NNBCs. The temperature elevation of NNBCs was modulated from 11.7 to 38.1 °C at a concentrations of 50 to 200 µg mL^−1^, which had a comparable temperature elevation to that of Nb_2_C (Figure 2c,d). These results indicate that the presence of Ni nanoparticles has a minimal impact on the photothermal conversion capability of Nb_2_C. In addition, the temperature of NNBCs rapidly increased to 71.6 °C at 2.5 W cm^−2^ within 5 min, while only a slight temperature elevation was observed at 0.5 W cm^−2^ (Figure 2f). Therefore, the temperature increment of the NNBCs displayed concentration‐ and power‐density‐dependent patterns upon irradiation by the 1064 nm laser. Furthermore, we can appropriately regulate the temperature of the NNBCs solutions by regulating the concentration of the NNBCs or power density of the applied NIR‐II laser. Moreover, the NNBCs demonstrated a photothermal conversion efficiency of 28.4% when exposed to the 1064 nm laser (Figure 2h).^[^ ^18^ ^]^ To further evaluate the laser‐induced photothermal stability of the NNBCs, the recovery temperature variations of the NNBCs upon exposure to an NIR laser were detected. As shown in Figure 2g, the temperature elevation of the NNBCs presented no apparent fluctuations during five on/off processes. These results confirmed the excellent photothermal conversion capability to achieve satisfactory photothermal elimination of bacteria in vitro and in vivo.

Encouraged by the efficient photothermal conversion of NNBCs, we further investigated the hyperthermia‐induced CO_2_ production capacity of the NNBCs upon exposure to an NIR laser. Nb_2_C and Ni/Nb_2_C without BC were chosen as control samples. As depicted in Figure 2i, Nb_2_C, Nb_2_C@BC, Ni/Nb_2_C, and NNBCs were dispersed in pre‐boiled deionized water to remove dissolved CO_2_ and then irradiated by a 1064 nm laser, followed by centrifugation to obtain the supernatant. Because CO_2_ can react with calcium hydroxide (Ca(OH)_2_) in water to produce a calcium carbonate (CaCO_3_) precipitate, an aqueous solution containing Ca(OH)_2_ was introduced into the supernatant for CO_2_ detection. After the addition of Ca(OH)_2_, no precipitation was observed in the Nb_2_C and Ni/Nb_2_C solutions, whereas a gray‐white CaCO_3_ precipitate appeared in the Nb_2_C@BC and NNBC solutions, indicating that Nb_2_C@BC and NNBCs could activate the dissociation of BC to efficiently produce CO_2_. Considering that dissolved CO_2_ can acidify the solution, we used the acid‐base indicator, bromothymol blue (BTB), to study the pH of the supernatant. After the addition of BTB, the colors of the Nb_2_C@BC and NNBC solutions changed from green to yellow as the reaction time increased (Figure 2j,k). This phenomenon indicates the relatively low pH of the supernatant in these groups, further validating the thermal‐effect‐induced CO_2_ production under 1064 nm laser irradiation (Figure 2l). Furthermore, we monitored and recorded of CO_2_ generation for different durations of 1064 nm laser irradiation. CO_2_ generation exhibited time‐dependent trends when subjected to irradiation with a 1064 nm laser (Figure S3, Supporting Information). Notably, the NNBCs exhibited a similar efficiency in CO_2_ generation as Nb_2_C@BC, suggesting that both Nb_2_C@BC and NNBCs can facilitate the dissociation of BC for the efficient production of CO_2_ (Figure S4, Supporting Information).

Inspired by the efficient CO_2_ production catalyzed by the NNBC‐mediated hyperthermia effect, we further investigated whether NNBCs could be used as photothermal catalysts to convert CO_2_ to CO. Based on previous studies, real‐time CO generation was monitored using a CO probe.^[^ ^19^ ^]^ In the presence of palladium chloride (PdCl_2_), a non‐fluorescent CO probe exhibited intense CO fluorescence (Figure 2m). Subsequently, the CO probe and PdCl_2_ were added to a NNBC solution, and the mixture was exposed to the 1064 nm laser. The fluorescence spectrum of the NNBCs showed significant fluorescence enhancement in the range of 510−516 nm, suggesting efficient CO generation upon laser irradiation (Figure 2n). In addition, we investigated the CO generation of various nanosheets, when subjected to 1064 nm laser irradiation. As displayed in Figure S5 (Supporting Information), no notable increase in fluorescence was observed in the other groups, suggesting the photothermal catalytic potential of the NNBCs. Since myoglobin (Mb) binds to CO to form carboxy‐myoglobin (CO‐Mb), we further confirmed the CO production of the NNBCs under 1064 nm laser irradiation using a standard Mb assay (Figure 2o).^[^ ^20^ ^]^ The UV–vis spectrum of Mb exhibited a characteristic peak at 560 nm, while the two apparent absorption peaks of CO‐Mb at 540 nm and 580 nm gradually appeared with increasing irradiation time, indicating the successful generation of CO. Moreover, the normalized absorbance profile of CO‐Mb in various treatment groups indicated that the CO generation in the NNBC *plus* NIR group was higher compared to that in the other groups (Figures S6 and S7, Supporting Information).

**Figure 3:**
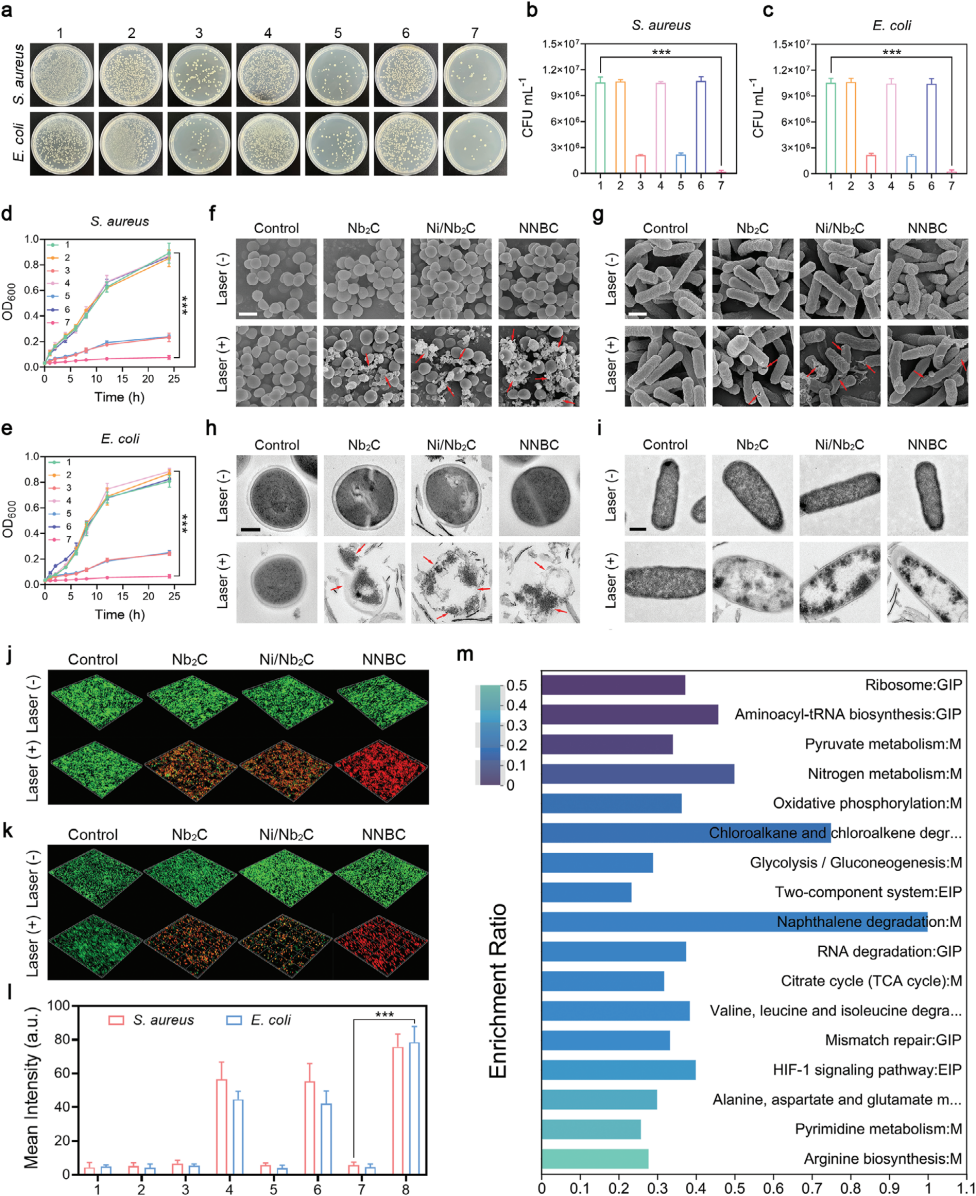
Antibacterial performance of NNBCs in vitro. a) Photographs of agar plates showing the growth of *E. coli* and *S. aureus* receiving various treatments. b,c) The bactericidal activity of various treatments against b) *S. aureus* and (c) *E. coli* (n = 5). d,e) Growth curves of d) *S. aureus* and e) *E. coli* receiving various treatments (1: control; 2: Nb_2_C; 3: Nb_2_C + NIR; 4: Ni/Nb_2_C; 5: Ni/Nb_2_C + NIR; 6: NNBC; 7: NNBC + NIR; n = 5). f,g) SEM images of f) *E. coli* and g) *S. aureus* in various treatment groups (scale bar = 1 µm). (h, i) TEM images of h) *E. coli* (scale bar = 500 nm) and i) *S. aureus* in different treatment groups (scale bar = 200 nm). j,k) Live/dead staining of j) *S. aureus* and k) *E. coli* after diverse treatments (Green fluorescence: live bacteria stained by SYTO9; red fluorescence: dead bacteria stained by PI). l) The corresponding semiquantitative analysis of CLSM images in j,k) (n = 3). m) Enrichment analysis of KEGG from the DEGs in the NNBC + NIR treated group compared to the control group. All data are presented as the mean ± SD, *p < 0.05, **p < 0.01, ***p < 0.001.

Based on the satisfactory photothermal properties and CO generation of NNBCs, their antibacterial ability was further studied. We selected typical infectious microorganisms, such as Staphylococcus aureus (*S. aureus*) and Escherichia coli (*E. coli*) to represent Gram‐positive and Gram‐negative bacteria, respectively. Subsequently, we designated seven different treatment groups, including control, Nb_2_C, Nb_2_C + NIR, Ni/ Nb_2_C, Ni/Nb_2_C + NIR, NNBC, and NNBC + NIR. Colony‐forming units (CFUs) in the various treatment groups were assessed using standard plate counting and corresponding photographs. The results indicate that numerous bacterial colonies survived in the control, Nb_2_C, Ni/Nb_2_C, and NNBC groups (**Figure** **3**a). Compared to the Nb_2_C + NIR and Ni/Nb_2_C + NIR groups, the NNBC + NIR group exhibited better antibacterial efficacy when exposed to 1064 nm laser due to the combination of hyperthermia effect and CO generation (Figure 3b,c). Based on the CFU reduction in *S. aureus*, the antibacterial efficiencies were 79.05%, 81.32% and 98.17%. Similarly, in terms of the reduction in the CFU of *E. coli*, the antibacterial efficiencies of the Nb_2_C + NIR, Ni/Nb_2_C + NIR, and NNBC + NIR groups were 79.42%, 80.33%, and 97.72%, respectively.

Afterward, the bacterial eradication ability of the different groups after incubation with *S. aureus* and *E. coli* for 24 h was investigated by measuring the absorbance at 600 nm, and the corresponding growth curves were plotted.^[^ ^21^ ^]^ Significant increases in OD_600 nm_ of *S. aureus* and *E. coli* were observed in the control, Nb_2_C, Ni/Nb_2_C, and NNBC groups during the 24 h incubation period. However, the growth of *E. coli* and *S. aureus* in the Nb_2_C + NIR, Ni/Nb_2_C + NIR, and NNBC + NIR groups was completely inhibited, demonstrating that the NNBCs were ineffective in eliminating bacteria without NIR laser irradiation (Figure 3d,e). To further elucidate the antibacterial mechanism of NNBCs under 1064 nm laser irradiation, scanning electron microscopy (SEM) was utilized to examine the alterations in bacterial morphology after various treatments.

As presented in Figure 3f,g and *E. coli* and *S. aureus* in the control, Nb_2_C, Ni/Nb_2_C, and NNBC groups exhibited an intact clubbed shape and a smooth surface, respectively. However, the bacterial morphology changed significantly with the introduction of the NIR laser. SEM images from the NNBC + NIR treatment group clearly illustrated the compromised integrity and altered structure of the bacterial walls in both *E. coli* and *S. aureus*. This observation indicated that NNBCs effectively eradicated the bacteria by destroying the bacterial walls under 1064 nm laser exposure. Additionally, the enlarged TEM images displayed the significant changes in the bacteria, such as decreased intracellular density, morphological changes, and rupture of bacterial structures, implying a severe loss of cellular integrity and distinct leakage of cytoplasmic contents (Figure 3h,i). Subsequently, the antibacterial performance of NNBCs against *E. coli* and *S. aureus* under 1064 nm laser illumination was further studied using SYTO9 and propidium iodide (PI) assays, and the results of live‐dead bacterial staining were monitored by confocal laser scanning microscopy (CLSM).^[^ ^22^ ^]^ Compared to the other treated groups, CLSM images of the NNBC + NIR group showed an obvious red fluorescence signal, indicating that NNBCs exerted the strongest antibacterial performance under 1064 nm laser exposure (Figure S8, Supporting Information). Confocal 3D reconstruction images of live and dead staining of bacteria in the NNBC + NIR group exhibited extensive red fluorescence, consistent with the CLSM images (Figure 3j−l).

Furthermore, RNA‐seq transcriptomics was used to investigate the underlying mechanism of the bactericidal performance of NNBCs under 1064 nm laser irradiation. As depicted in Figures S9 and S10 (Supporting Information), compared with the control group, the NNBC + NIR group showed 420 upregulated and 295 downregulated differentially expressed genes (DEGs). In addition, a comprehensive enrichment analysis of the Kyoto Encyclopedia of Genes and Genomes (KEGG) pathway was performed to identify the most affected pathways. As depicted in Figure 3m, the tricarboxylic acid cycle (TCA) pathway and pyrimidine metabolism pathways associated with energy metabolism were highly involved in the downregulated enrichment pathway, indicating that NNBCs exhibited a significant effect on bacterial metabolism under 1064 nm laser irradiation. Similarly, gene ontology (GO) annotation analysis revealed the alteration of the metabolic process pathways primarily in the control and NNBC + NIR groups (Figure S11, Supporting Information).^[^ ^23^ ^]^ The aforementioned results collectively reveal that NNBCs possess superior antibacterial performance, which can be attributed to the hyperthermia effect and CO produced by NNBC‐mediated PTT. This holds promising potential in effectively combating antibiotic resistance.

**Figure 4:**
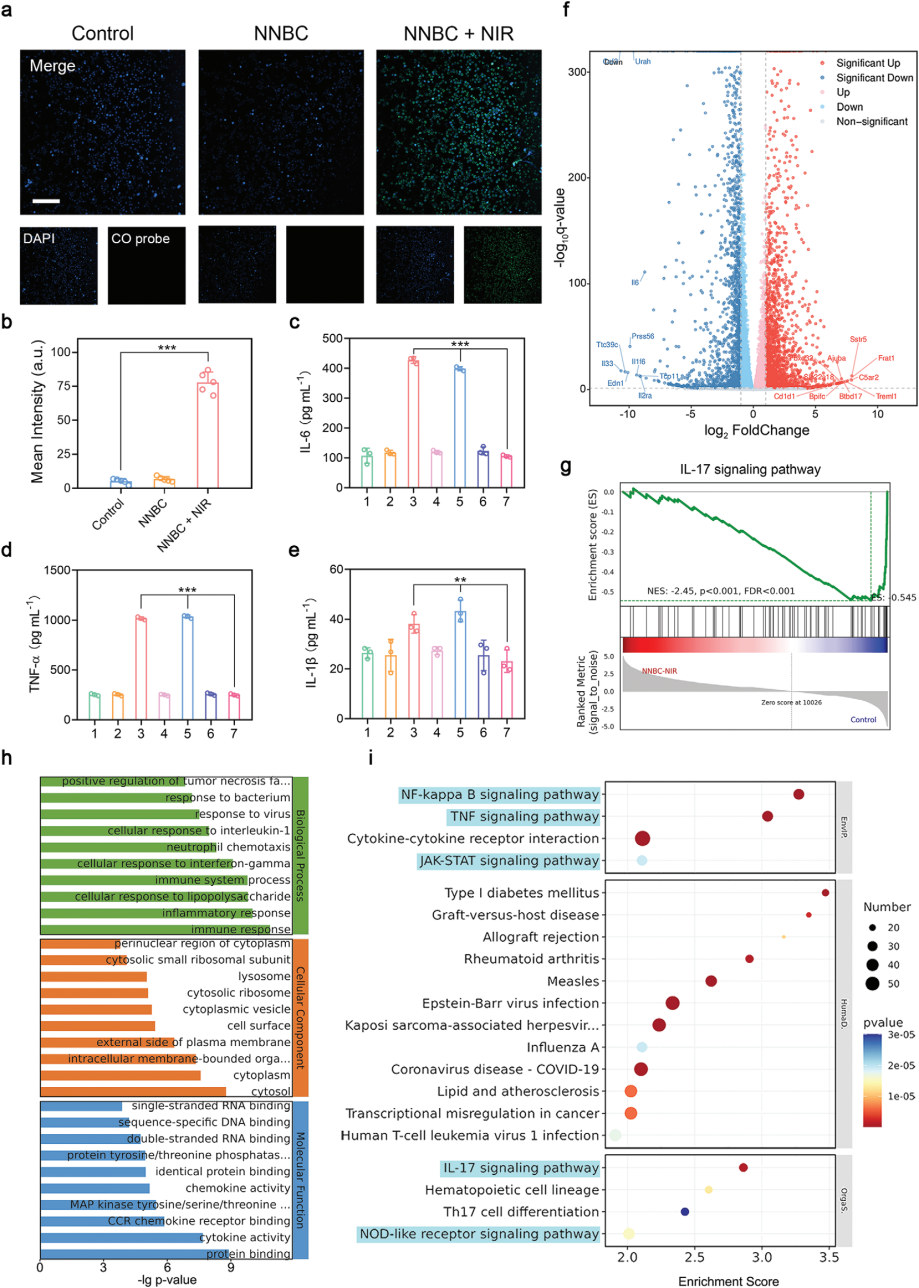
Potential mechanisms of the anti‐inflammatory performance of NNBCs under 1064 nm laser irradiation by transcriptome high throughput sequencing analysis. a) Fluorescence images of CO in RAW 264.7 macrophages after various treatments (blue and green fluorescence: RAW 264.7 macrophages stained by DAPI and CO fluorescent probe, respectively; scale bar = 100 µm) and b) the corresponding semi‐quantitative analysis of the green fluorescence intensities (n = 5). c) IL‐6, d) TNF‐α, and e) IL‐1β levels of RAW 264.7 macrophages from diverse treatment groups (1: control; 2: Nb_2_C; 3: Nb_2_C + NIR; 4: Ni/Nb_2_C; 5: Ni/Nb_2_C + NIR; 6: NNBC; 7: NNBC + NIR; n = 3). f) Comparative volcano plots illustrating the up‐regulated (red) and down‐regulated (blue) genes (cut‐off logarithm 10 (fold change) ≥2.0) of RAW 264.7 cells between the NNBC + NIR and control groups by RNA‐Seq analysis. g) Topological graph of the “IL‐17 signaling pathway” mediated by NNBC + NIR in the Reactome data. h,i) Enrichment analysis of h) GO and i) KEGG from the differential expression of genes in NNBC + NIR treated RAW 264.7 cells compared to the control ones (cut‐off logarithm10 (fold change) ≥2.0). All data are presented as the mean ± SD, *p < 0.05, **p < 0.01, ***p < 0.001.

Before evaluating the in vitro anti‐inflammatory effects of NNBCs under the 1064 nm laser illumination, it is essential to confirm whether NNBCs are cytotoxic to normal cells. Human kidney‐2 (HK‐2) cells and human umbilical vein endothelial cells (HUVECs) were used to assess the biosafety of NNBCs. After incubation with NNBCs for 24 and 48 h, cell viability was estimated using cell counting kit‐8 (CCK‐8) assays. As displayed in Figure S12 (Supporting Information), the toxicity of the NNBCs toward HK‐2 cells and HUVECs was negligible after 48 h of incubation, suggesting that the NNBCs are highly biocompatible. Thereafter, to determine whether NNBCs could efficiently produce CO in living cells, a mouse macrophage cell line (RAW 264.7) was selected as the cell model and incubated with NNBCs under both non‐laser and 1064 nm‐laser incubated conditions. Compared to the negative, control, and NNBC groups, obvious green fluorescence enhancement was observed in the NNBC + NIR group, indicating the efficient CO generation in cells under 1064 nm laser exposure owing to the NNBCs (**Figure** **4**a,b). Since the hyperthermic effect of NNBCs under NIR laser illumination led to a severe inflammatory response, we further investigated whether the produced CO could effectively mitigate the inflammatory response. Lipopolysaccharide (LPS), derived from Gram‐negative bacteria, were utilized as a stimulant to induce inflammation. After exposure to LPS, RAW 264.7 macrophages exhibited a spindle‐shaped morphology with visible pseudopodia, reminiscent of the cellular characteristics observed in the Nb_2_C + NIR and Ni/Nb_2_C + NIR groups (Figure S13, Supporting Information). However, the morphology of the RAW 264.7 macrophages displayed no discernible alterations after treatment with NNBC + NIR. These results substantiate that the CO produced can attenuate inflammation and evade macrophage activation. Moreover, the expression of the pro‐inflammatory cytokines including interleukin‐6 (IL‐6), tumor necrosis factor α (TNF‐α), and interleukin‐1β (IL‐1β), on the RAW 264.7 macrophages was detected by an enzyme‐linked immunosorbent assay (ELISA) to estimate the inflammatory capacity after various treatments.^[^ ^24^ ^]^ As presented in Figure 4c−e, significant overproduction of IL‐6, TNF‐α, and IL‐1β was observed in the Nb_2_C + NIR and Ni/Nb_2_C + NIR groups, indicating that hyperthermia effect caused a remarkable pro‐inflammatory response. In contrast, the secretion of IL‐6, TNF‐α and IL‐1β in the NNBC + NIR group was minimally affected, illustrating that the inflammatory response caused by hyperthermia effect was alleviated in the presence of CO.

An RNA‐seq analysis of RAW 264.7 macrophages was performed to reveal the underlying anti‐inflammatory mechanism of NNBCs under NIR laser irradiation and to investigate the significantly different mRNAs of the control and NNBC + NIR groups. Principal component analysis (PCA) that the transcriptomic characteristics of the NNBC + NIR group were extremely distinct from those of the control group (Figure S14, Supporting Information). In Figure 4f, volcano plots display the DEGs according to filtering criteria. Among these DEGs, there were 2145 upregulated and 1853 downregulated genes in the NNBC + NIR group. GO enrichment analysis was conducted on these DEGs to better understand the biological processes altered by NNBC + NIR laser illumination. As presented in Figure 4h and Figure S15 (Supporting Information), these downregulated DEGs were involved in inflammatory, cellular, and immune responses, suggesting suppression of the inflammatory response. A bubble map of the down‐regulated DEGs enriched in the KEGG pathway, shown in Figure 4i, was used to investigate the pathways that promote the anti‐inflammatory response in vitro. NF‐kappa B, TNF, and IL‐17 signaling factors play critical roles in regulating the inflammatory response.^[^ ^25^ ^]^ The NF‐kappa B, TNF, and IL‐17 signaling pathways were highly involved in the first 20 down‐regulated enhancement pathways, confirming that NNBCs efficiently regulate the inflammatory response by adjusting various inflammation‐related signaling pathways. In addition, Janus tyrosine kinase signal transducer and activator of transcription (JAK‐STAT) signaling pathway and nucleotide‐binding oligomerization domain‐like (NOD‐like) receptor signaling pathways, involved in the regulation of inflammatory responses, were also significantly downregulated.^[^ ^26^ ^]^ Furthermore, gene set enrichment analysis (GSEA) was performed to verify the downregulation of relevant genes in these signaling pathways. As displayed in Figure 4g and Figure S16 (Supporting Information), the analysis results of the NF‐kappa B, TNF, and IL‐17 signaling pathways were in accordance with the KEGG analysis. These findings validate that NNBCs combined with NIR laser irradiation can effectively inhibit inflammation by regulating the inflammation‐related response pathways.

Motivated by the remarkable antibacterial and anti‐inflammatory abilities of NNBCs in vitro under 1064 nm laser irradiation, skin wound models with *S. aureus* infection were used to further evaluate their antibacterial and anti‐inflammatory effects in vivo. First, the in vivo toxicity of the NNBC was evaluated to verify its biosafety. Balb/c mice were randomly assigned into four groups and treated with elevated doses of NNBCs. There were no significant variations in the body weight of the mice during treatment, illustrating the high biocompatibility of NNBCs (Figure S17, Supporting Information). 15 days after injection, the mice were euthanized, and their main organs and blood samples were gathered for histological and hematological analyses, respectively. No obvious pathological changes and inflammatory cell‐infiltration were found in the hematoxylin and eosin (H&E) staining images of the main organs, and the corresponding blood parameters were corroborated to be normal (Figures S18−S21, Supporting Information). These results confirm that NNBCs are highly biocompatible for further utilization in vivo.

**Figure 5:**
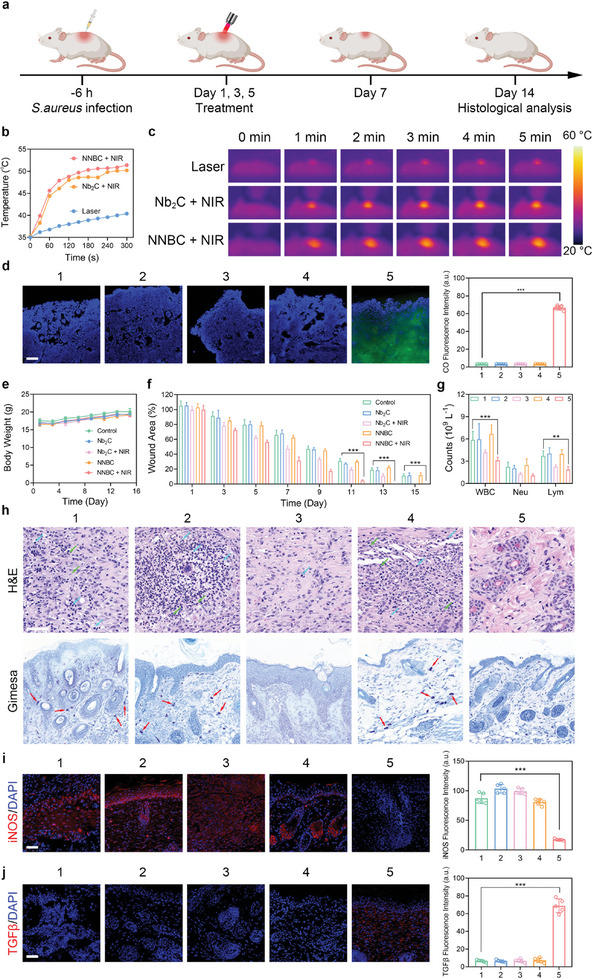
In vivo experiment on the antibacterial ability of NNBCs in a skin wound model. a) Schematic illustration of the mouse model establishment and therapeutic process. b) Photothermal heating curves in various treatment groups and c) the corresponding thermographic images. d) CO generation in mouse skin wounds after various treatments (blue fluorescence: DAPI; green fluorescence: CO probe; scale bar = 50 µm; n = 5). e) Changes in the body weights of the mice from different treatment groups (n = 5). f) Quantitative analysis of the residual wounded areas after different treatments (n = 5). g) Blood parameters from various treatment groups, including white blood cell (WBC), neutrophils (Neu), and lymphocytes (Lym) (n = 5). h) H&E and Giemsa staining of the skin tissue in the wound areas from various treatment groups (green arrow: Neu; blue arrow: Lym; red arrow: bacteria). i) Inducible nitric oxide synthase (iNOS, M1‐type), and j) transforming growth factor *β* (TGF‐*β*, M2‐type) immunofluorescence staining of the skin tissue in the wound areas and the corresponding semi‐quantitative analysis. (1: control; 2: Nb_2_C; 3: Nb_2_C + NIR; 4: NNBC; 5: NNBC + NIR; scale bar = 50 µm; n = 5). All data are presented as the mean ± SD, *p < 0.05, **p < 0.01, ***p < 0.001.

A round, full‐thickness cutaneous wound with a diameter of 8 mm was constructed using a surgical blade, and the wound was immediately inoculated with a *S. aureus* suspension. After 6 h, infected mice were stochastically divided into five groups to receive various treatments (**Figure** **5**a). Before investigating the antibacterial properties in vivo, the wound areas of the control, Nb_2_C + NIR, and NNBC + NIR groups were under the 1064 nm laser illumination for 5 min. The temperature profiles and thermal images of the wound areas are shown in Figure 5b,c. The temperature of the wound area in the NNBC + NIR group increased from 35 to 51.4 °C within 5 min, which was sufficient to kill bacteria. However, for the same irradiation time, the temperature increase in the control group was negligible, and the temperature elevation caused by Nb_2_C was limited. Subsequently, CO production in the skin wounds area was monitored after various treatments. An intensive green fluorescence in the NNBC + NIR group illustrated the efficient generation of CO, but negligible fluorescence enhancement was observed in other treatment groups (Figure 5d). These encouraging results demonstrate that the hyperthermic effect and CO produced by the NNBCs in the skin wound area have a high potential to eradicate bacterial pathogens, alleviate associated inflammation, and accelerate wound healing.

Furthermore, we recorded the body weights of the mice, skin wound areas, and representative macroscopic photographs of skin tissues from the various treatment groups during the 14‐day treatment period. Mice from different treatment groups experienced a slight increase in body weight throughout the treatment period, indicating the high biocompatibility of NNBCs under the 1064 nm laser irradiation (Figure 5e). On day 1, the successful construction of the model was evidenced by prominent redness in all groups. Compared to the other treatment groups, the NNBC + NIR group exhibited the most rapid healing of infected wounds, achieving non‐inflammatory closure by day 9, and complete recovery by day 13. These findings demonstrate the outstanding antibacterial activity and superior therapeutic performance of NNBCs under NIR‐II laser illumination (Figure 5f; Figure S22, Supporting Information). After 14 days of treatment, the mice were sacrificed, and skin tissues from the wound areas, major organs, and blood samples were collected for in vivo anti‐inflammatory investigation. As depicted in Figure 5g, blood indices associated with inflammatory responses, such as white blood cells (WBC), neutrophils (Neu), and lymphocytes (Lym), were also analyzed. The blood parameters in the NNBC + NIR group were lower than those in the other groups. In addition, the bacterial count in each treatment group on day 15 was determined by standard CFU counting, and the results indicated that the bacterial count in the NNBC + NIR group was the lowest, revealing that the NNBC + NIR group had the best wound healing performance (Figure S23, Supporting Information). Histological analysis showed that the NNBC + NIR group had the least infiltration of inflammatory cells and the fewest bacteria (Figure 5h).

To further investigate the immune microenvironment of the skin wound areas, immunofluorescence analyses of inducible nitric oxide synthase (iNOS) and transforming growth factor‐β (TGF‐β) expression were performed (Figure 5i,j). Due to the potent antibacterial and anti‐inflammatory performance of the hyperthermia effect in combination with CO generation, the NNBC + NIR group exhibited the lowest iNOS expression and highest TGF‐β expression compared to the other treatment groups, indicating that the macrophages tended to polarize toward M2‐type for favorable wound healing after 15 days of treatment. Additionally, no distinct pathological changes or detrimental reactions were observed in the H&E staining of the major organs in the NNBC + NIR group, further verifying the high biocompatibility of the NNBCs (Figure S24, Supporting Information).

**Figure 6:**
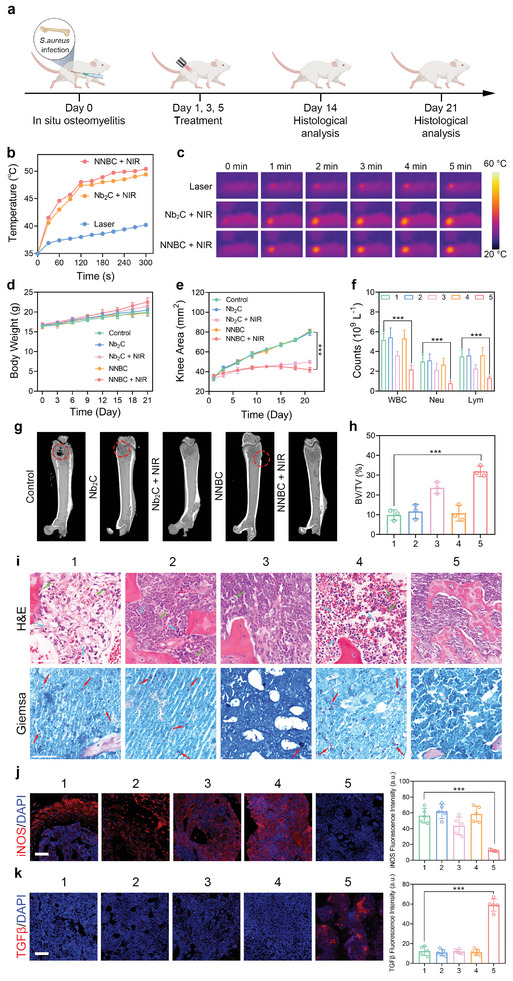
Treatment of osteomyelitis with NNBCs in vivo. a) Schematic illustration of the in situ osteomyelitis model establishment and treatment process. b) Photothermal heating curves, and c) the corresponding thermographic images from different treatment groups. d) Body weights of the mice, e) the average knee areas of the infected knees, and the f) blood parameters, including WBC, Neu and Lym, from various treatment groups (n = 5). g) Mircocomputed tomography (MCT) images, and h) the corresponding bone volume/total volume (BV/TV) results of the infected legs (n = 3). i) H&E and Gimesa staining of the infected bone tissue (Green arrow: Neu; Blue arrow: Lym; Red arrow: bacteria). j) iNOS and k) TGF‐*β* immunofluorescence staining of the infected bone tissue and the corresponding semi‐quantitative analysis (1: control; 2: Nb_2_C; 3: Nb_2_C + NIR; 4: NNBC; 5: NNBC + NIR; scale bar = 50 µm; n = 5). All data are presented as the mean ± SD, *p < 0.05, **p < 0.01, ***p < 0.001.

Given the satisfactory efficacy of NNBCs in eliminating bacteria and alleviating inflammation in the skin wound model, we further investigated their therapeutic efficacy of NNBCs in treating osteomyelitis under NIR‐II laser exposure. A *S. aureus* suspension was injected into the left femur marrow cavity of the mice to establish an osteomyelitis model (**Figure** **6**a). Subsequently, the osteomyelitis mice were randomly divided into five groups. To confirm whether NNBCs could produce a hyperthermic effect in the bone marrow cavity, the photothermal effect of NNBCs under NIR‐II laser irradiation was investigated. As depicted in Figure 6b,c, the femur temperature of the mice treated with NNBCs + NIR‐II, along with laser irradiation, increased rapidly and reached 50.4 °C within 5 min, suggesting the high capability of the NNBCs in triggering photothermal conversion in vivo.

After verifying the effective photothermal performance of the osteomyelitis model, mice from different groups were subjected to various treatments, and the knee areas and body weights of the mice were measured every three days. From the knee area curves and photographs of the surgical sites on days 1 and 21, severe swelling and purulent knee joints were observed in the infected legs of the mice in the control, Nb_2_C, and NNBC groups. In contrast, no distinct variation was observed in the legs of the mice in the Nb_2_C + NIR and NNBC + NIR groups (Figure 6e). In addition, the body weights of the mice from the Nb_2_C + NIR and NNBC + NIR groups increased steadily, whereas the body weights of the mice from the groups without NIR‐II laser exposure experienced slower body weight gain owing to the presence of infection (Figure 6d). After three weeks of treatment, blood samples were gathered for routine blood tests to analyze the inflammatory cells. Similar to the results of the skin wound model, the counts of the WBC, Neu, and Lym in the NNBC + NIR group were lower than those in the other treatment groups, indicating that NNBCs effectively alleviated systemic inflammatory responses in vivo under NIR‐II laser irradiation (Figure 6f).

Next, infected bone tissues from all treatment groups were harvested and analyzed by micro‐computerized tomography (MCT) to study the anti‐inflammatory effect of the NNBCs in the osteomyelitis model under NIR‐II laser irradiation. Based on the MCT reconstruction images depicted in Figure 6g, significant bone destruction occurred in the control, Nb_2_C, and NNBC groups, whereas the NNBC + NIR group exhibited the smallest defect size compared to other treatment groups. Additionally, the bone volume/total volume (BV/TV) values in each treatment group were calculated based on the MCT reconstruction images. Among all treatment groups, the NNBC + NIR group exhibited the highest BV/TV value, which agreed with the smallest area of bone defects (Figure 6h).

Furthermore, we assessed the microbial changes in infected bone tissues with osteomyelitis from various treatment groups. A CFU assay using harvested bone tissues showed a marked reduction in the number of bacterial colonies in the NNBC + NIR group (Figure S25, Supporting Information). H&E and Gimesa staining of the infected bone tissues was performed to assess the therapeutic effect of NNBCs under NIR‐II laser exposure. Well‐structured bone tissues were apparently observed in the NNBC + NIR group, whereas a large number of inflammatory cells and bacterial infiltration were detected in the bone tissues from the control, Nb_2_C, and NNBC groups (Figure 6i). Additionally, immunofluorescence staining of macrophages was performed to evaluate the anti‐inflammatory effects of CO produced by NNBCs. As expected, the NNBC + NIR group exhibited the least iNOS expression and most TGFβ expression, which were beneficial for the bone regeneration (Figure 6j,k). Therefore, under 1064 nm laser illumination, NNBCs can not only be used as excellent antibacterial nanomaterials to destroy bacteria through the hyperthermia effect, but also to alleviate the accompanying inflammation and thus accelerate tissue regeneration.

## 3 Conclusion

In summary, a photothermal catalytic CO generator called NNBCs was constructed to simultaneously inhibit bacterial proliferation and relieve inflammatory infections. Under a 1064 nm laser irradiation, NNBCs achieved efficient photothermal CO_2_ catalysis for CO generation. Moreover, the hyperthermia effect triggered by NNBCs caused the decomposition of BC to generate CO_2_ near the nanoplatforms, thereby augmenting CO production under 1064 nm laser exposure. Owing to their prominent hyperthermic effect and efficient CO generation, in vivo assessments using an osteomyelitis model confirmed the effective inhibition of bacterial growth and attenuation of the inflammatory response induced by the photothermal effect associated with the NNBCs. This study provides a therapeutic paradigm to achieve photothermal catalytic CO generation under 1064 nm laser exposure and bridges the gap between photothermal CO_2_ catalysis and nanoplatforms to simultaneously suppress bacterial growth and relieve inflammatory infection.

## 4 Experimental Section

### Materials

All the chemicals were used without additional purification steps. Niobium aluminum carbide powder (Nb_2_AlC) with a purity greater than 95% and particle size of 400 mesh was obtained from Forsman Scientific (Beijing) Co., Ltd. Hydrofluoric acid (HF) with a concentration of 49% in H_2_O, an ammonium hydroxide solution (NH_3_•H_2_O) with a concentration of 28%, and tetramethylammonium hydroxide pentahydrate (C_4_H_13_NO•5H_2_O, TMAH) with a purity of 97% were purchased from Shanghai Yuanye Bio‐Technology Co., Ltd. Additionally, Ni(NO_3_)_2_•6H_2_O and polyvinylpyrrolidone (PVP), with a molecular weight of 360 000, were purchased from Sigma–Aldrich (Shanghai) Cp., Ltd. Mouse IL‐6 ELISA Kit (U96‐1511E), Mouse TNF‐α ELISA Kit (U96‐3112E), and Mouse IL‐1β ELISA Kit (U96‐1494E) were purchased from YOBIBIO (Shanghai, China).

### Fabrication of Nb_2_C

Production of few‐layer Nb_2_C involves a revised two‐step exfoliation technique. First, 5 g of Nb_2_AlC powder was dissolved in 30 mL of an aqueous HF solution (49 wt.%) under magnetic stirring, at room temperature. After four days of stirring, the remaining solids were gathered through centrifugation and washed multiple times with deionized (DI) water to eliminate any remaining HF. The purified specimen was subsequently dispersed in 20 mL of a TMAH solution (50 wt.%) with intense mixing at ambient temperature for three days. Following a three‐day exposure to TMAH, the resulting material was gathered through centrifugation and washed multiple times with DI water and ethanol, and to obtain the few‐layer Nb_2_C, which was suspended in DI water.

### Fabrication of Ni/Nb_2_C

116.3 mg of Ni(NO_3_)_2_•6H_2_O, 200 mg of PVP, and 125.5 mg of few‐layer Nb_2_C were combined in 20 mL of water and magnetically stirred for 10 min. Afterward, 120 µL of NH_3_•H_2_O was added to the reaction mixture under magnetic stirring for 30 min. The mixed solution was placed in a Teflon‐lined autoclave and heated at 150 °C for 24 h. After cooling to ambient temperature, black solid Ni(OH)_2_/Nb_2_C was gathered through centrifugation, rinsed multiple times with water and ethanol, and then dehydrated in a vacuum oven. The Ni/Nb_2_C product was obtained by reducing the dried powder at 300 °C for 3 h in an H_2_ atmosphere.

### Fabrication of NNBCs

50 mg of NH_2_‐PEG‐DPA was mixed with a Ni/Nb_2_C aqueous solution (1 mg mL^−1^, 10 mL) and stirred overnight at room temperature. The mixture was centrifuged and washed with DI water and ethanol. The obtained product was distributed in ethanol. Subsequently, 50 mg of FeCl_3_•6H_2_O was added to the ethanol solution. After stirring overnight, the mixture was centrifuged and washed with DI water and ethanol, to obtain Fe^3+^‐modified Ni/Nb_2_C. Afterward, 1 mL of a NH_4_HCO_3_ solution (5 mM) was added to the Fe^3+^‐modified Ni/Nb_2_C ethanol solution every hour in a chilled bath. This process was repeated five times. Finally, the product was centrifuged and washed with ice water to obtain the NNBCs.

### Characterization

TEM images were captured using an FEI Tecnai F20 transmission electron microscope, while energy dispersive X‐ray spectroscopy (EDS) spectra were acquired using a JEM‐2100 F electron microscope. A Thermo Scientific K‐alpha X‐ray photoemission spectrometer was used to collect spectral data via X‐ray photoelectron spectroscopy (XPS). Measurements of particle size distribution and ζ potential were conducted using a Zetasizer Nanoseries device. XRD patterns were acquired with a Rigaku D/Max‐2200 PC XRD instrument, utilizing Cu Kα radiation, 40 mA current, and 40 kV voltage. A UV‐3600 Shimadzu UV–vis‐NIR spectrometer was used to collect the UV–vis‐NIR absorption spectra. CLSM images were captured using an FV1000 Olympus confocal laser scanning microscope.

### In Vitro Photothermal Effect

To measure the photothermal conversion performance of NNBCs, aqueous solutions (100 µL) containing different concentrations of Nb_2_C, Ni/Nb_2_C, or NNBCs (0–200 µg mL^−1^) were introduced into 96‐well plates and exposed to a 1064 nm NIR laser at a power density of 1.5 W cm^−2^ for 5 min. Furthermore, aqueous solutions of NNBCs were exposed to a 1064 nm laser at varying power densities (0.5, 1.0, 1.5, 2.0, and 2.5 W cm^−2^). An infrared (IR) thermal camera was used to record the temperature and obtain thermal images. The photothermal stability of the NNBCs exposed to the NIR laser was assessed by monitoring the temperature changes during five heating and cooling cycles.

### CO_2_ Detection

DI water was preheated to eliminate naturally dissolved CO_2_. Following this, various liquid solutions with Nb_2_C, Nb_2_C@BC, Ni/Nb_2_C, or NNBCs (200 µg mL^−1^) were subjected to a 1064 nm laser irradiation (1.5 W cm^−2^). After 5 min of exposure, the aqueous solution was centrifuged at 10 000 rpm for 15 min). The supernatant was collected and added to 0.5 mL of a calcium hydroxide (Ca (OH)_2_) aqueous solution (1 mg mL^−1^). The mixture was then centrifuged to assess CaCO_3_ precipitation. Then, BTB was added to the supernatant. Subsequently, the pH of the solution was determined, and photographs of the solution were recorded.

### CO Detection

A mixture of NNBCs (50 µg), CO probe (5 µM), and PdCl_2_ (5 µM) in 1 mL of aqueous solution was used for CO detection. The fluorescence spectra of the mixture were measured every minute for 5 min while being exposed to the 1064 nm laser irradiation (1 W cm^−2^). The CO probe was excited at a wavelength of 490 nm.

### Myoglobin Assay

A myoglobin solution with a concentration of 0.5 mg mL^−1^ in phosphate‐buffered saline (PBS) was degassed with N_2_ gas bubbling. Then, sodium dithionite (0.1%) was added to create a deoxygenated solution with a myoglobin concentration of 27 µM. Next, 50 µg of NNBCs was introduced, followed by irradiation with a 1064 nm laser. The corresponding UV–vis spectra of the samples were recorded.

### In Vitro Antibacterial Assay

*E. coli* (ATCC25922) and *S. aureus* (ATCC6538) served as the Gram‐negative and ‐positive bacterial strains, respectively. A single colony of either *E. coli* or *S. aureus* was grown in liquid Luria–Bertani (LB) broth with agitation at 37 °C for 12–16 h. Bacterial population densities were assessed by measuring absorbance at 600 nm. 200 µL of the bacterial suspension with 10^6^ CFU mL^−1^ was placed in 96‐well plates with Nb_2_C, Ni/Nb_2_C, or NNBCs. The plates were then irradiated with/without 1064 nm laser irradiation. After various treatments, the bacteria were cultivated on agar plates at 37 °C. A control group that did not receive any treatment was included.

### SEM Observation

SEM was used to examine the morphological alterations of *S. aureus* and *E. coli* following exposure to PBS, Nb_2_C, Ni/Nb_2_C, and NNBCs, with or without irradiation. The antibacterial properties were evaluated according to the aforementioned protocols, which involved rinsing the treated bacteria with PBS twice and then fixing them overnight with 2.5% glutaraldehyde at 4 °C. The bacteria were washed with sterile PBS and sequentially dehydrated using gradient concentrations of ethanol for 10 min. The resulting samples were deposited onto a silicon wafer and dried overnight under vacuum. The specimens were coated with platinum and subjected to SEM observation.

### TEM Observation

*S. aureus* and *E. coli* were inoculated and grown in a tryptic soy broth (TSB) medium at 37 °C. Afterward, the bacteria were then thinned in sterile PBS to a concentration of 10^7^ CFU mL^−1^. A combination of 5 mL of the bacterial solution and 5 mL of PBS, Nb_2_C, Ni/Nb_2_C, or NNBCs (100 µg mL^−1^) was blended, followed by exposure to a 1064 nm laser. This solution was then incubated for an additional 10 min at 37 °C. The microbes were gathered, rinsed three times using PBS, and then preserved overnight at 4 °C using a 2.5% glutaraldehyde solution. The samples were dehydrated using a series of ethanol solutions of increasing concentrations and rinsed three times with acetone. The resulting samples were then incubated in 500 µL of an epoxy solution with epoxy/acetone ratios of 1 and 3 for 1 h and 3 h, respectively. The samples were then immersed in a fresh resin solution (100%) for ≈36 h. Subsequently, an additional amount of resin (250 µL) was introduced, and the samples were cured at 70 °C for 2 days. Finally, the cured samples were cut into sections with a thickness of 70 nm using a Leica UC7 ultramicrotome equipped with a diamond knife. The obtained slices were placed on copper grids for TEM observation.

### Live/Dead Staining Assay

200 µL of the bacterial suspension was introduced into 96‐well plates with PBS, Nb_2_C, Ni/Nb_2_C, or NNBCs (100 µg mL^−1^) and irradiated with/without 1064 nm laser. Subsequently, the bacteria were stained with a SYTO9/PI live/dead bacterial double stain kit. After three washes with PBS, the bacteria were examined using CLSM.

### In Vitro Metabonomic Studies of S. aureus

*S. aureus* was cultivated with NNBCs and subsequently subjected to 1064 nm laser irradiation. Additionally, a control group without any treatment was included. Prior to total RNA extraction, 2 mL of lysozyme (2 mg mL^−1^) was introduced into the bacteria to facilitate cell wall lysis. Afterwards, complete RNA was ioslated using the RNeasy Mini Kit, and the resulting samples were examined for gene expression.

### In Vitro Cytotoxicity Evaluation of NNBCs

HUVECs and HK‐2 cells were selected as the cell models to evaluate in vitro cytotoxicity. HUVECs or HK‐2 cells were seeded into 96‐well plates and incubated overnight. Subsequently, different concentrations of NNBCs were administered to the cells. After 24 or 48 h of incubation, cell viability was assessed using a cell counting kit‐8 assay.

### Intracellular CO Detection

For intracellular CO detection, RAW 264.7 macrophages were incubated with NNBCs for 6 h. Subsequently, the culture medium was replaced with a fresh medium, and the cells were irradiated with the 1064 nm laser. Afterward, the cells were exposed to a combination of the CO probe and PbCl_2_ for 30 min, rinsed with PBS, and examined by CLSM.

### Detection of Intracellular Cytokine Production

To quantify the levels of IL‐6, TNF‐α, and IL‐1β in the RAW 264.7 macrophages, cells were planted in 24‐well plates at a density of 10^5^ cells per well and incubated for 24 h. The cells were then treated with PBS, Nb_2_C, Ni/Nb_2_C, or NNBCs. After incubating the cells at 37 °C for 4 h, they were then subjected to the 1064 nm laser irradiation. The plates were then incubated at 37 °C for 24 h. Subsequently, 50 µL of the cell culture supernatant was collected, and the levels of IL‐6, TNF‐α, and IL‐1β in each well were measured with an ELISA kit.

### In Vitro Gene Transcription Analysis in RAW 264.7 Macrophages

Transcriptome high throughput sequencing was conducted on the mRNAs extracted from the RAW 264.7 macrophages of the NNBC + NIR group. Additionally, a control group without any treatment was included. Following various treatments, the RAW 264.7 macrophages were sent to Oebitech Co., Ltd. (Shanghai, China) for gene expression analysis.

### Experimental Animals

Male 4‐week‐old Balb/c mice were obtained from Zhongshan Hospital, Fudan University. All the experiments and procedures were approved under the guidelines of the Animal Ethics Committee of Zhongshan Hospital, Fudan University (2023‐058).

### In Vivo Biosafety Evaluation

Healthy mice were randomized into four groups and intravenously administered various doses of NNBCs. The body weights of the mice were measured every three days. Following a 15‐day injection period, the mice were anesthetized, and the major organs and blood samples were gathered for analyzing the blood and tissue characteristics.

### Hemolysis Test

Hemolysis of NNBCs was assessed using recently collected mouse blood. The blood was centrifugated at 3000 rpm for 15 min at 4 °C. Subsequently, the supernatant was removed, and the remaining red blood cells (RBCs) were gathered and rinsed three times with PBS. Various quantities of the NNBC solution were mixed with 1 mL of the RBC (2%) suspension, followed by incubation at 37 °C for 3 h. The supernatant from each group was collected after centrifugation at 1500 rpm for 15 min to determine absorbance at 450 nm. DI water and PBS were employed as the positive and negative controls, respectively.

### In Vivo Antibacterial Evaluation Using Mouse Cutaneous Wound Models

Male Balb/c mice, aged 4–6 weeks, were utilized for in vivo assessment. The Animal Ethics Committee of Zhongshan Hospital, Fudan University, oversaw compliance with all animal experiments and procedures. Twenty‐five mice were randomly assigned to the following five groups, with five mice per group: 1) Control, 2) Nb_2_C, 3) Nb_2_C + NIR, 4) NNBC, and 5) NNBC + NIR. After anesthetizing the mice with isoflurane, an 8 mm^2^ full‐thickness skin wound was created on the dorsal skin by excision. 20 µL of the bacterial suspension (10^7^ CFU mL^−1^) was inoculated into the wound tissues of the mice. Six hours after injection, the wounds were exposed to 20 µL of PBS, Nb_2_C, or NNBCs with/without exposure to the 1064 nm laser. Throughout the treatment period, the body weights and wound areas of the mice from the various treatment groups were recorded., Wounds photographs were captured using a digital camera on days 1, 5, 9 and 13. Infected wound tissues and vital organs were gathered by the 15th day. Subsequently, the injured wound tissues and major organs were collected and preserved in 4% paraformaldehyde for staining with H&E and Gimesa stains. Additionally, the skin wound tissues were stained with iNOS and TGF‐*β* to explore the inflammatory responses in various treatment groups.

### In Vivo Antibacterial Evaluation Using Mouse Osteomyelitis Models

Mice were anesthetized with 1% (w/w) pentobarbital (20 mg kg^−1^). Subsequently, the hair on the hind legs were removed, and the skin was disinfected. A small incision was made using a scalpel. A dental drill was then used to construct a cortical bone defect with a diameter of 1.4 mm^2^. Then, 50 µL of the *S. aureus* suspension was injected into the intramedullary canal through the bone defects to establish an in situ osteomyelitis model. The mice were randomly divided to the following five groups: Control, Nb_2_C, Nb_2_C + NIR, NNBC, and NNBC + NIR. After receiving different treatments, the weights and areas of infected knees were recorded. A caliper was used to longitudinally measure the knee area, which was then calculated using the formula, knee area = π × a × b, where a and b represented the lengths of the semi‐major and ‐minor axes, respectively. Infected femurs were collected by sacrificing the mice for histopathological examination. Infected bone tissues were stained with H&E and Gimesa staines. To study the inflammatory response, the infected bone tissues were subjected to iNOS and TGF‐*β* staining.

### Micro‐CT Analysis

The femurs of mice after different treatments were collected for Micro‐CT analysis. Scanning was conducted using an X‐ray voltage of 65 kVp, at an anode current of 385 µA. 3D Creator software was utilized to reconstruct the tomograms. New bone formation was evaluated by calculating the BV/TV ratio using MCT.

### Statistical Analysis

Data were presented as mean ± SD, and the significance of differences between the two groups was assessed using the student's two‐tailed *t* test or one‐way analysis of variance using GraphPad Prism 8 software (*p < 0.05; **p < 0.01; ***p < 0.001).

## Conflict of Interest

The authors declare no conflict of interest.

## Supporting information

Supporting Information

## Data Availability

The data that support the findings of this study are available from the corresponding author upon reasonable request.
