# Supplementary material for: Engineering Photothermal Catalytic CO2 Nanoreactor for Osteomyelitis Treatment by In Situ CO Generation
Source: Adv Sci (Weinh). 2024 Apr 22;11(25):2402256. doi: 10.1002/advs.202402256 (PMC11220635; doi:10.1002/advs.202402256)
Supplement: Supplementary file 1 — Supporting Information [file ADVS-11-2402256-s001.pdf]

ADVANCED SCIENCE
Open Access

Supporting Information

for *Adv. Sci.*, DOI 10.1002/advs.202402256

Engineering Photothermal Catalytic CO_2 Nanoreactor for Osteomyelitis Treatment by In Situ CO Generation

Fan Zhuang, Luxia Jing, Huijing Xiang, Cuixian Li, Beilei Lu, Lixia Yan, Jingjing Wang, Yu Chen* and Beijian Huang**

Supporting Information

Engineering Photothermal Catalytic CO_2 Nanoreactor for Osteomyelitis Treatment by In-Situ CO Generation

Fan Zhuang,[‡] Luxia Jing,[‡] Huijing Xiang, Cuixian Li, Beilei Lu, Lixia Yan, Jingjing Wang, Yu Chen*, and Beijian Huang**

Fan Zhuang, Luxia Jing, Cuixian Li, Beilei Lu, Lixia Yan, Jingjing Wang, and Prof. Beijian Huang
Department of Ultrasound, Zhongshan Hospital, Fudan University, and Shanghai Institute of Medical Imaging, Shanghai 200032, P. R. China.
E-mail: huang.beijian@zs-hospital.sh.cn

Prof. Huijing Xiang, and Prof. Yu Chen
Materdicine Lab, School of Life Sciences, Shanghai University, Shanghai 200444, P. R. China.
E-mail: xianghuijing@shu.edu.cn; chenyuedu@shu.edu.cn

Prof. Y. Chen
Oujiang Laboratory (Zhejiang Lab for Regenerative Medicine, Vision and Brain Health), Wenzhou Institute of Shanghai University, Wenzhou, Zhejiang 325088, P. R. China.

Prof. Y. Chen
Shanghai Institute of Materdicine, Shanghai 200051, P. R. China.

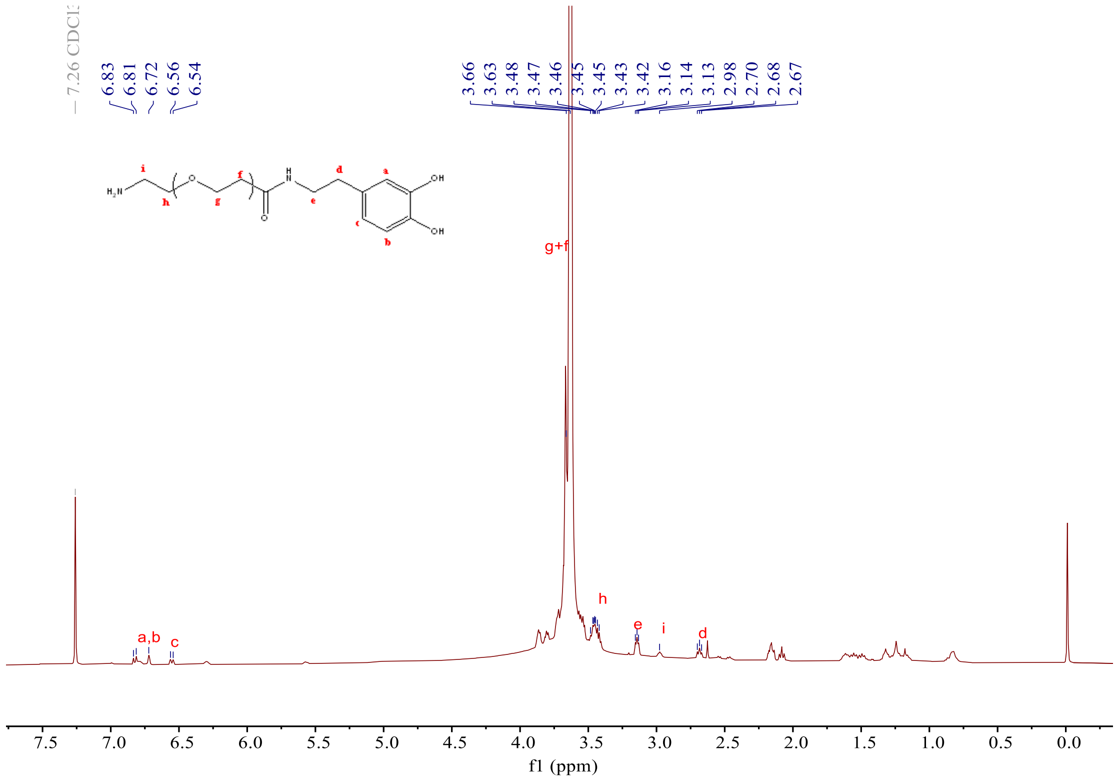

Figure S1. ^{1}H NMR spectra of NH_2-PEG-DPA (400 MHz, $CDCl_3$): δ 6.83 - 6.72 (m, 2H), 6.55 (d, J = 7.8 Hz, 1H), 3.84 - 3.63 (m), 3.49 – 3.38 (m, 2H), 3.14 (t, J = 4.7 Hz, 2H), 2.98 (m, 2H), 2.68 (t, J = 6.3 Hz, 2H).

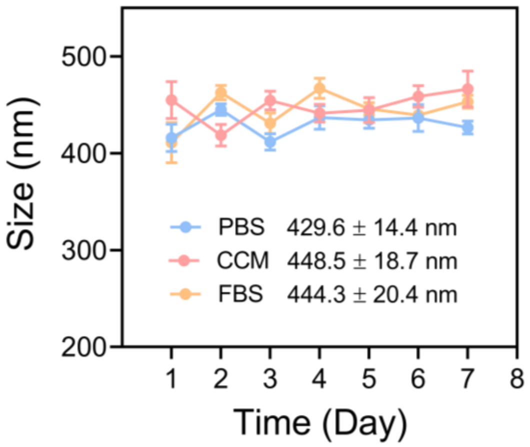

Figure S2. Hydrodynamic size distribution of NNBCs in various incubation solutions for seven days (n = 3). Data are presented as the mean ± SD.

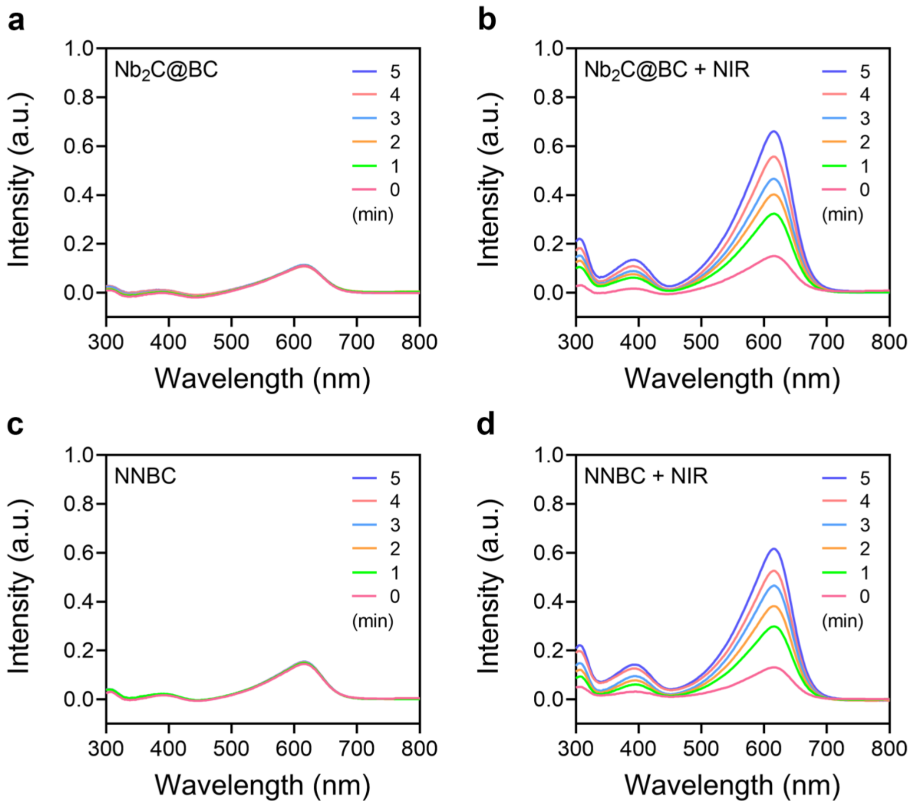

Figure S3. The UV-vis absorption spectra of various mixtures after addition of BTB in different treatment groups (a: Nb_2C@BC; b: Nb_2C@BC + NIR; c: NNBC; d: NNBC + NIR).

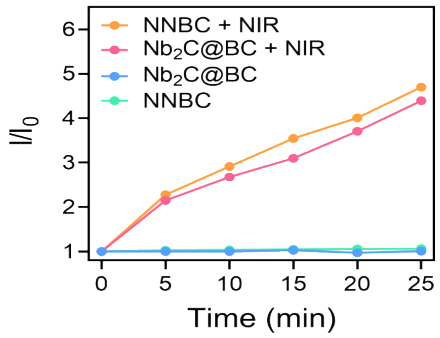

Figure S4. The normalized absorbance profile of BTB in different treatment groups.

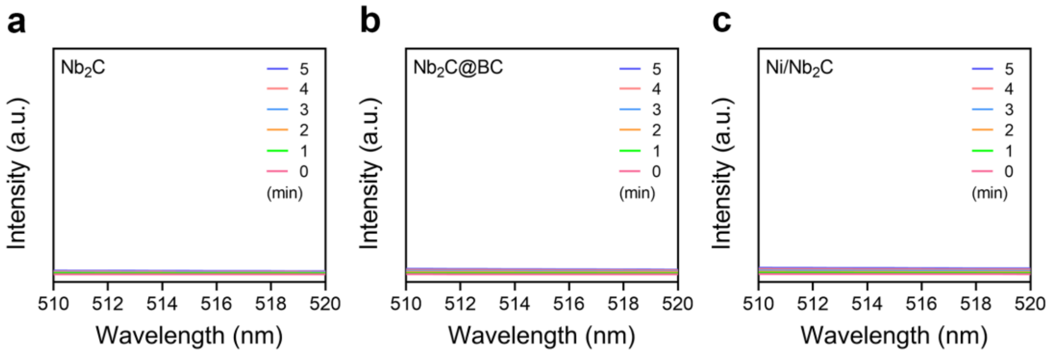

Figure S5. Fluorescent spectra changes of CO probe and $PdCl_2$ under 1064 nm laser irradiation in different treatment groups, including (a) Nb_2C, (b) Nb_2C@BC, and (c) Ni/Nb_2C.

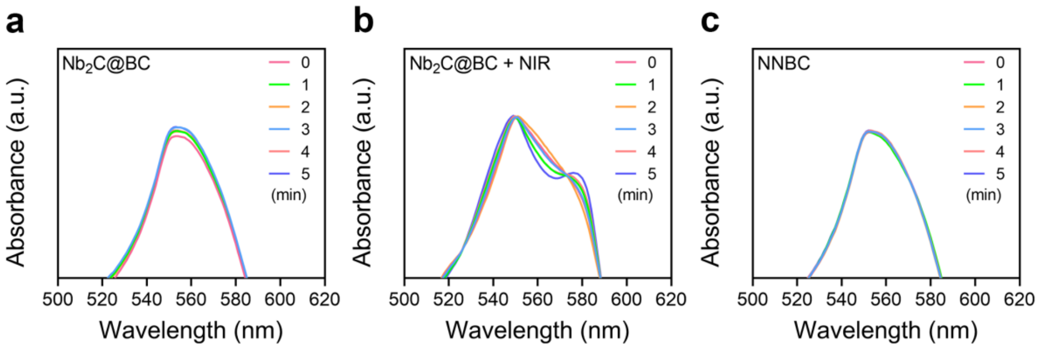

Figure S6. CO release profiles measured by myoglobin assay in various treatment groups, including (a) Nb_2C@BC, (b) Nb_2C@BC, and (c) NNBC.

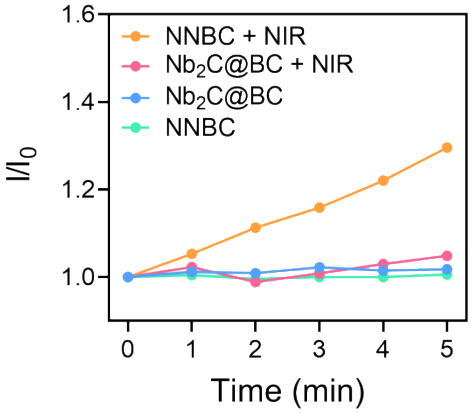

Figure S7. The normalized absorbance profile of CO-Mb in different treatment groups.

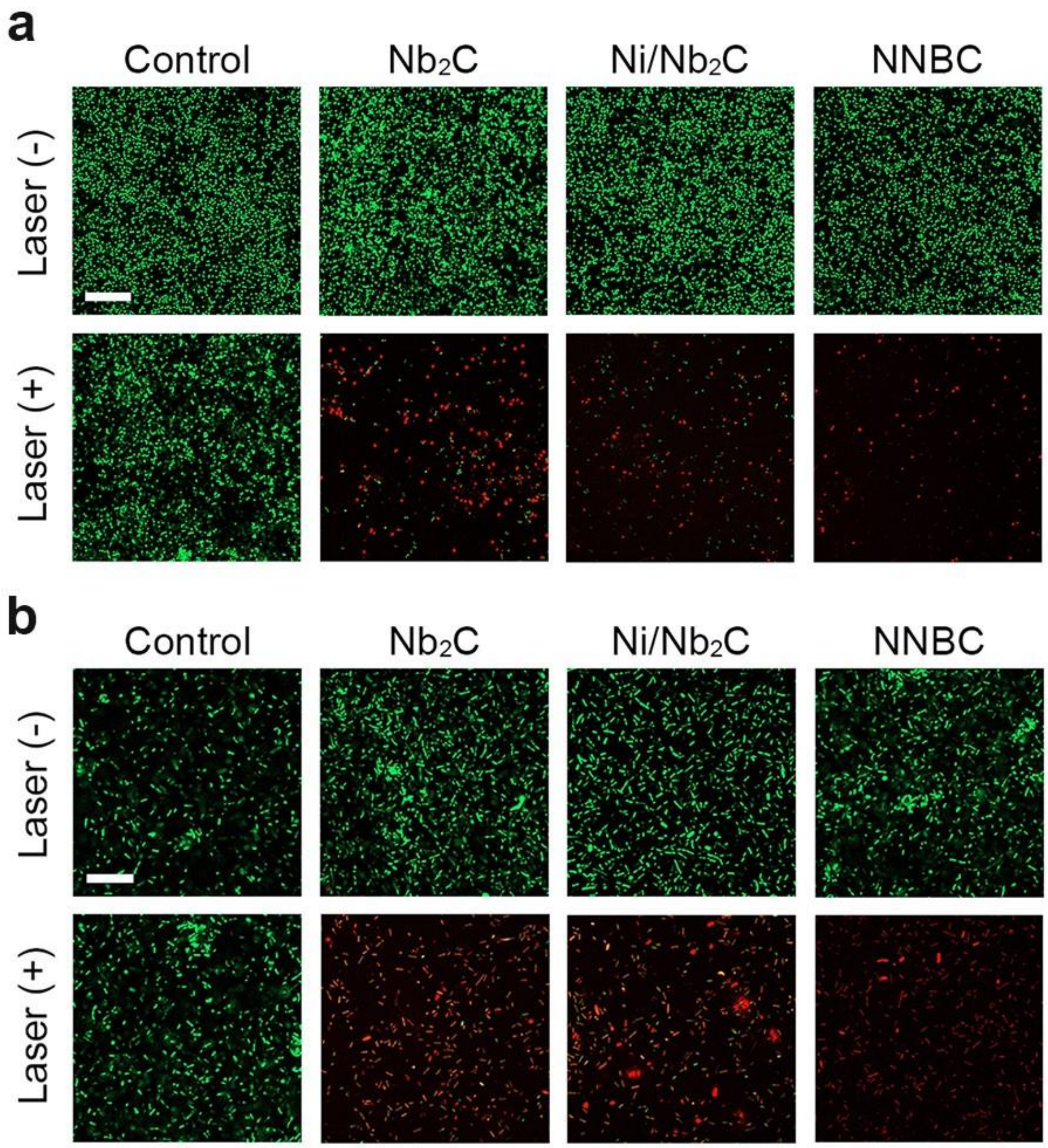

Figure S8. CLSM images of (a) *S. aureus* and (b) *E. coli* after diverse treatments (scale bar = 100 μm).

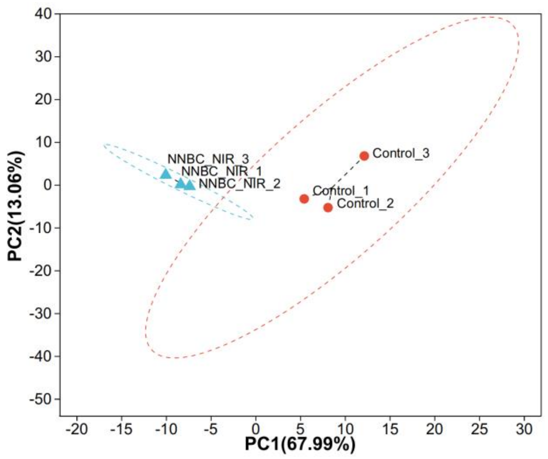

Figure S9. Principal component analysis (PCA) according to the differentially expressed mRNAs of *S. aureus* from the control and NNBC + NIR groups.

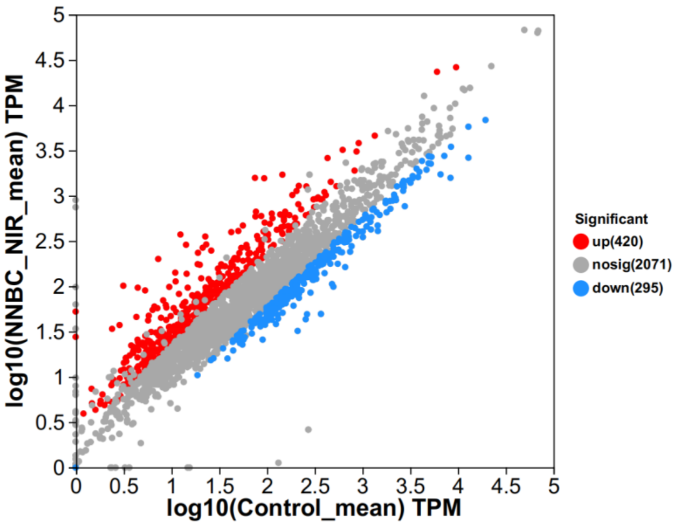

Figure S10. The scatter plot illustrating significantly different genes in the control and NNBC + NIR treatment groups.

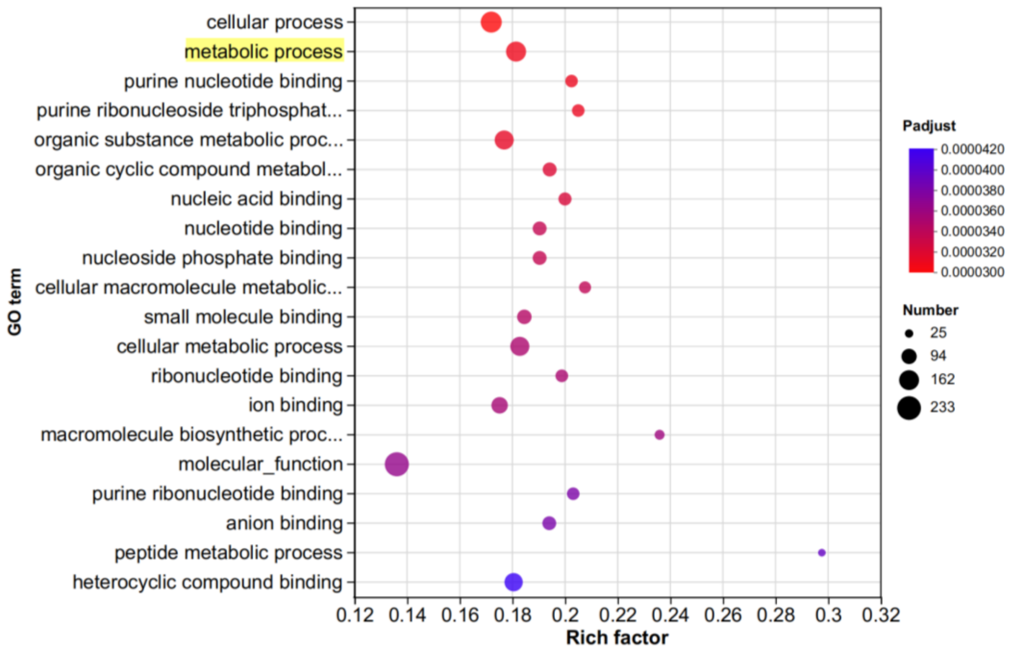

Figure S11. Gene ontology (GO) pathway enrichment of top 20 down-regulated pathways of *S. aureus* between control group and NNBC + NIR group.

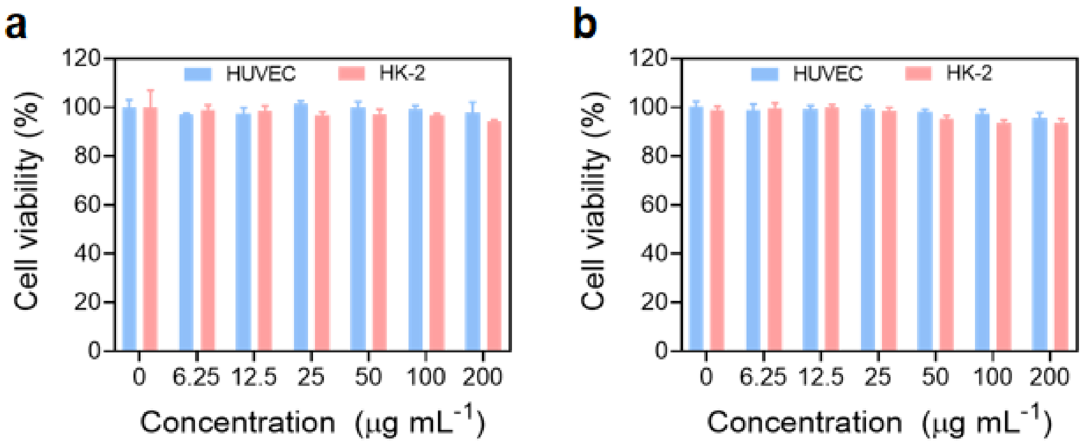

Figure S12. Cellular viabilities of HUVEC and HK-2 cells after cultured with increasing concentrations of NNBCs for (a) 24 h and (b) 48 h (n = 5). Data are presented as the mean ± SD.

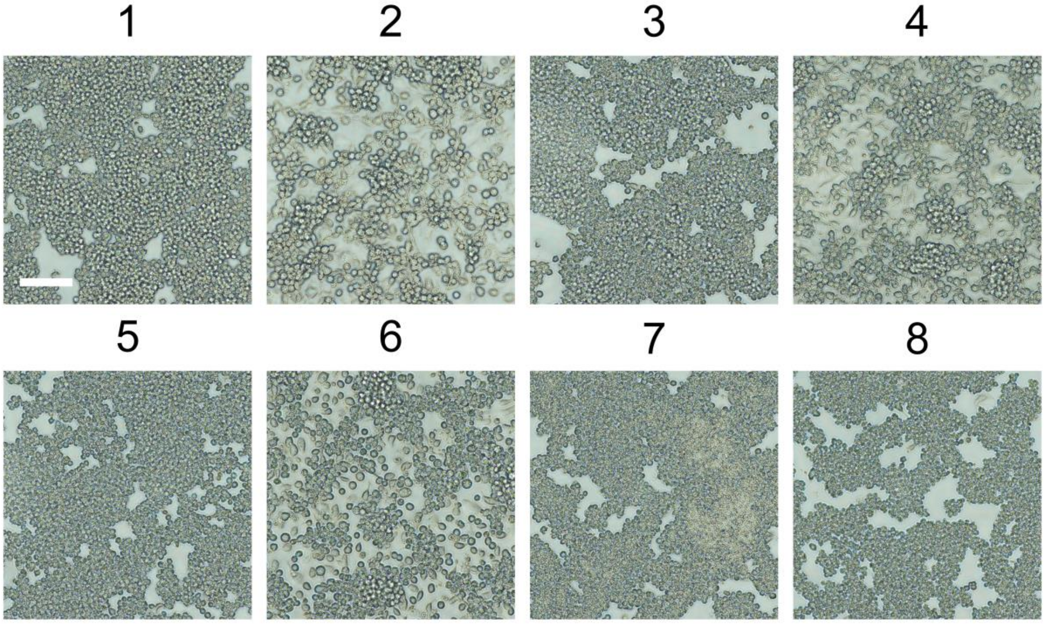

Figure S13. Cellular morphology of RAW 264.7 macrophages in different treatment groups (1: Control; 2: LPS; 3: Nb_2C; 4: Nb_2C + NIR; 5: Ni/Nb_2C; 6: Ni/Nb_2C + NIR; 7: NNBC; 8: NNBC + NIR; scale bar = 100 μm).

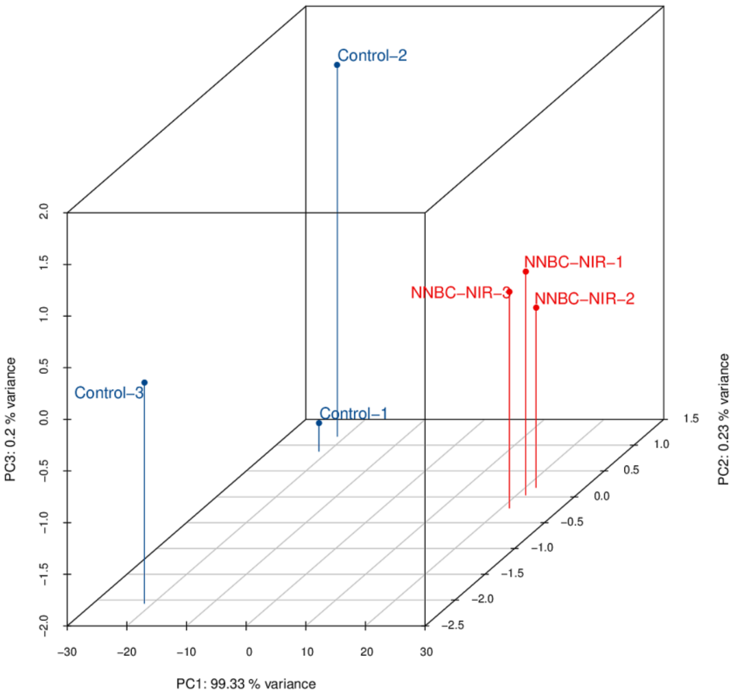

Figure S14. Principal component analysis (PCA) according to the differentially expressed mRNAs from the control and NNBC + NIR groups.

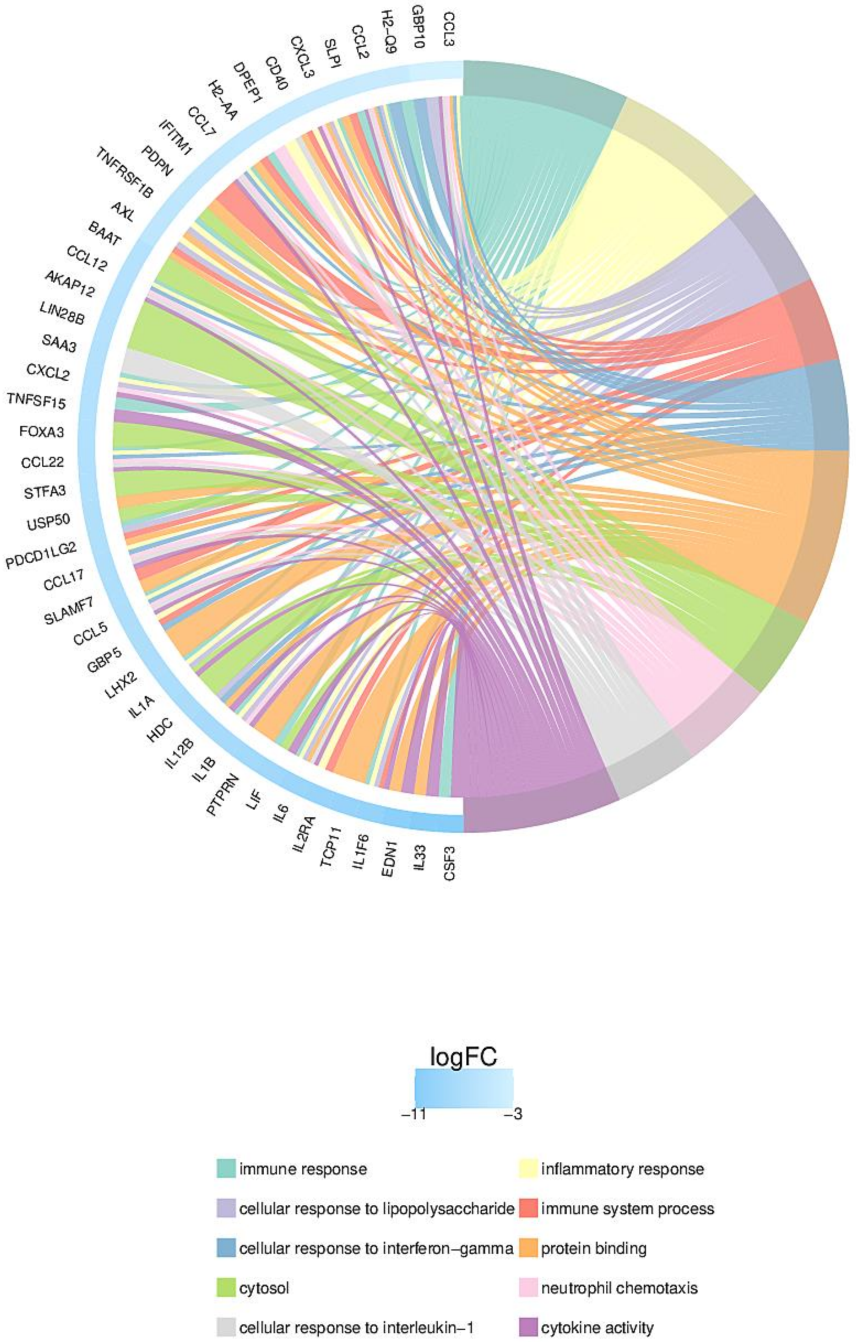

Figure S15. Chord diagram of enrichment analysis of GO from differential expression of genes in NNBC + NIR treated RAW 264.7 macrophages as compared to the control group.

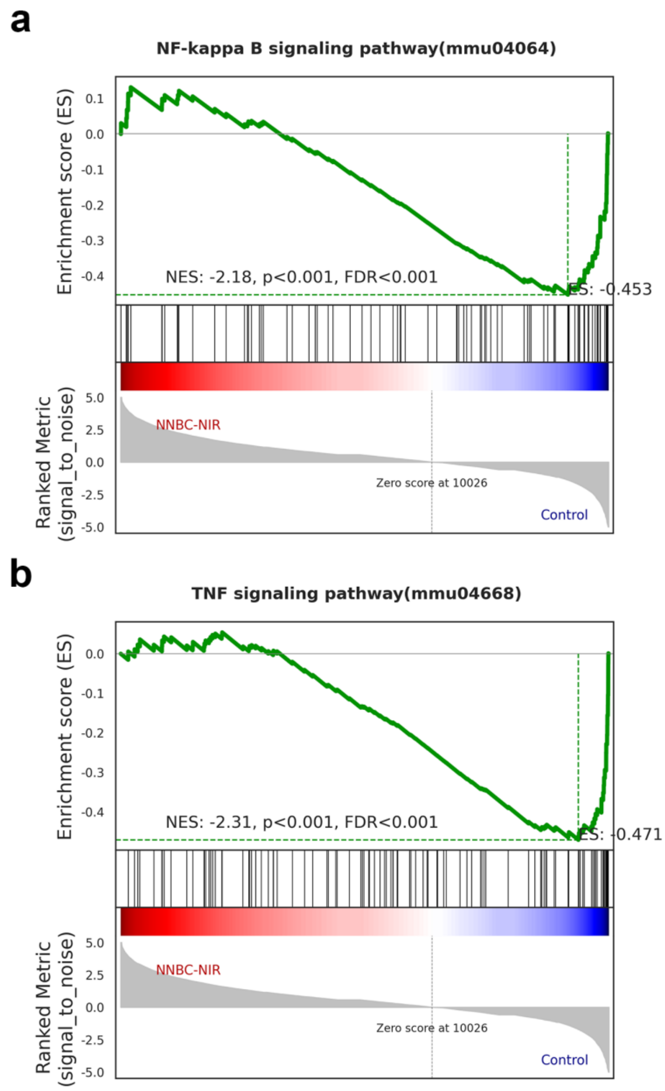

Figure S16. Topological graph of (a) “NF-kappa B signaling pathway” and (b) “TNF signaling pathway” mediated by NNBC + NIR” in the Reactome data.

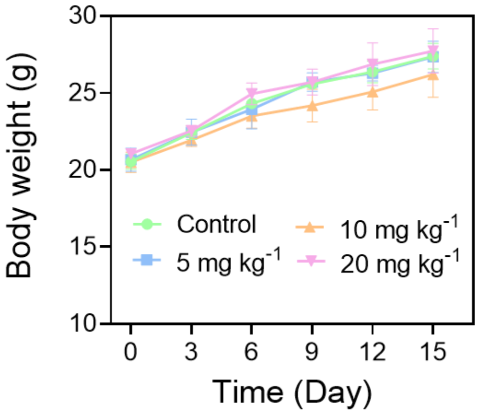

Figure S17. Body-weight variations of Balb/c mice after intravenous administration of diverse doses of NNBCs for 15 days (n = 5). Data are presented as the mean ± SD.

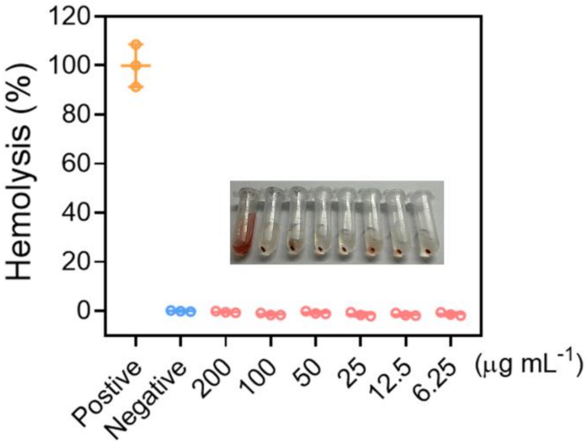

Figure S18. Hemolysis rate of NNBCs toward red blood cells after 3 h of incubation. DI water acted as a positive control; PBS acted as a negative control (n = 3). Data are presented as the mean ± SD.

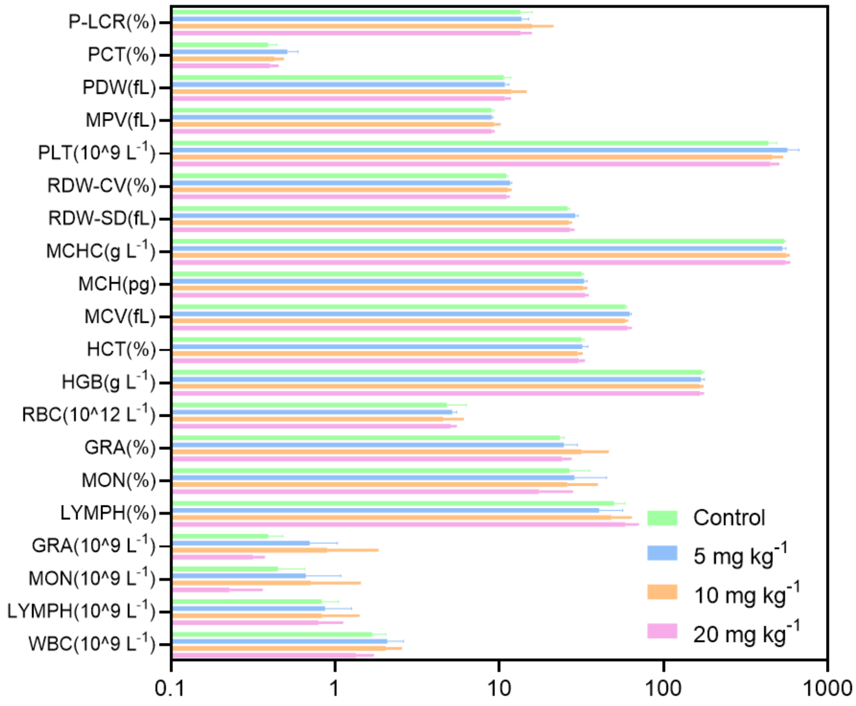

Figure S19. Hematological analysis of Balb/c mice after intravenous administration of various doses of NNBCs for 15 days. Blood routine index, including platelet-larger cell ratio (P-LCR), platelecrit (PCT), platelet distribution width (PDW), mean platelet volume (MPV), platelet (PLT), red cell distribution width-coefficient variation (RDW-CV), red cell distribution width-standard deviation (RDW-SD), mean corpuscular hemoglobin concentration (MCHC), mean corpuscular hemoglobin (MCH), mean corpuscular volume (MCV), hematocrit (HCT), hemoglobin (HGB), red blood cell (RBC), granulocyte (GRA), monocytes (MON), lymphocytes (LYMPH), white blood cell (WBC) (n = 5). Data are presented as the mean ± SD.

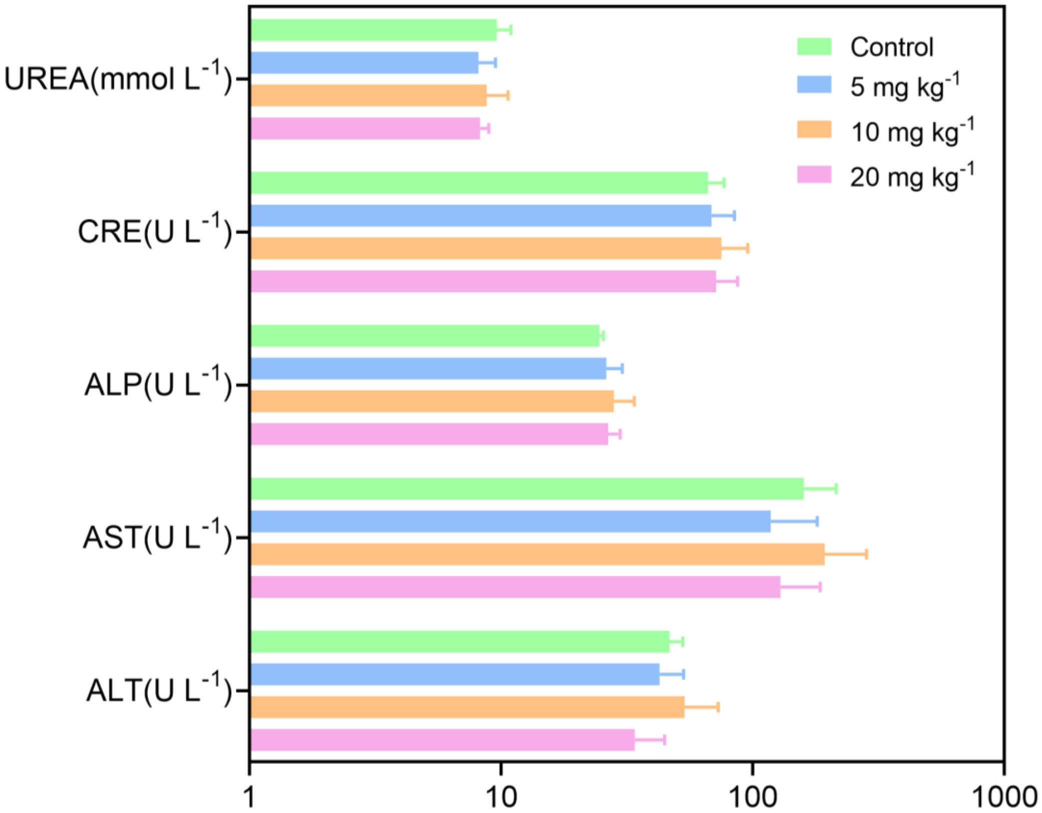

Figure S20. Hematological analysis of Balb/c mice after intravenous administration of different doses of MCA for 15 days. Biochemistry parameters, including urea, creatinine (CRE), alkaline phosphatase (ALP), aspartate transaminase (AST), and alanine transaminase (ALT) (n = 5). Data are presented as the mean ± SD.

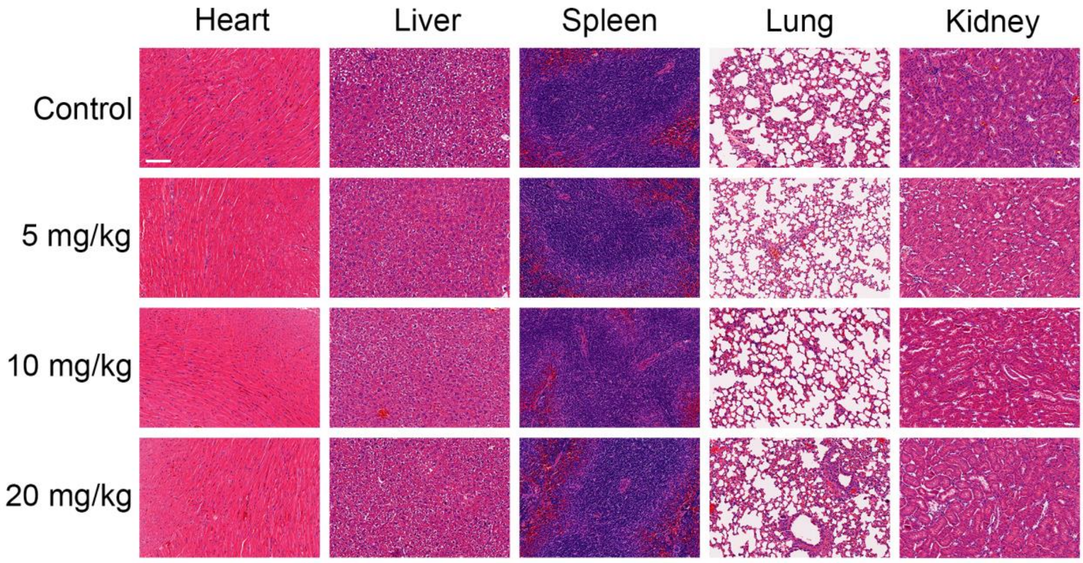

Figure S21. H&E staining images of major organs (heart, liver, spleen, lung, and kidney) collected from Balb/c mice after intravenous administration of various doses of NNBCs for 15 days (scale bar = 100 μm).

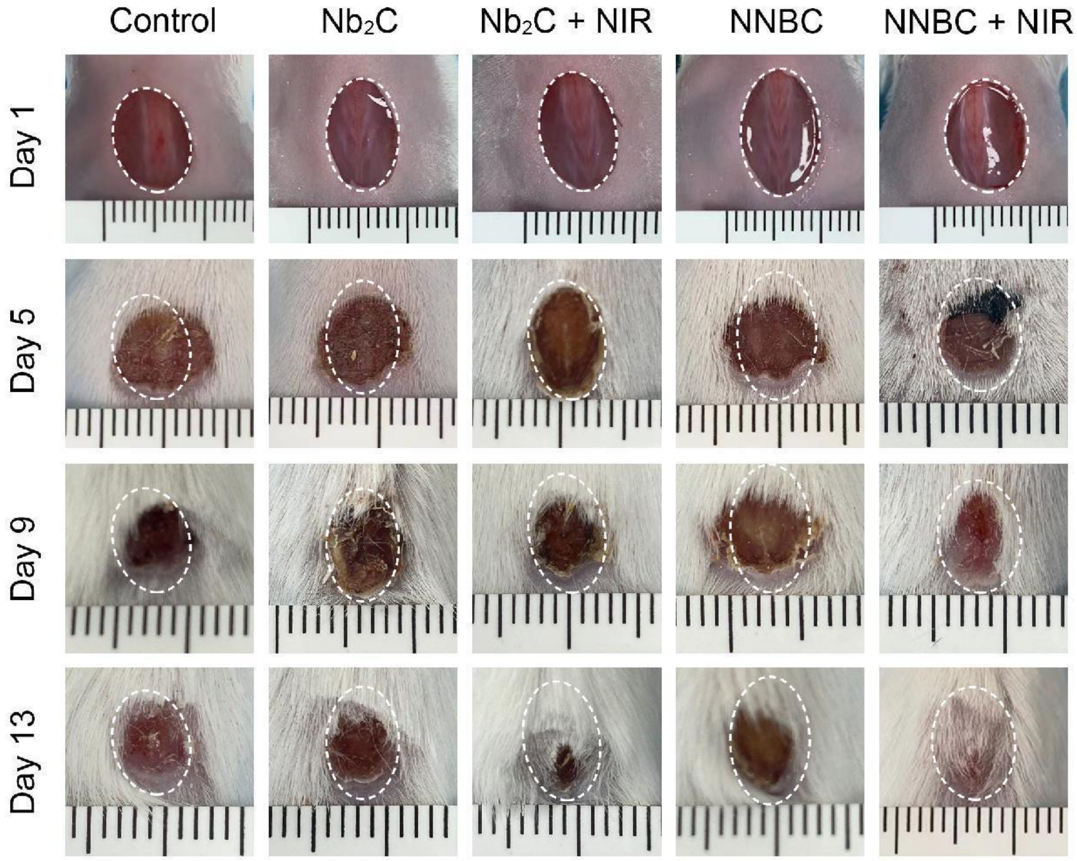

Figure S22. Photographs of cutaneous wounds of *S. aureus* infected mice receiving various treatments.

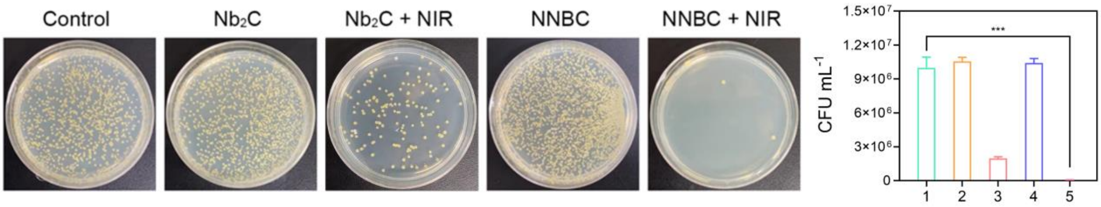

Figure S23. Photographs of *S. aureus* inoculated from skin wound tissues on agar plates and the corresponding bacterial activity of various treatments (1: Control; 2: Nb_2C; 3: Nb_2C + NIR; 4: NNBC; 5: NNBC + NIR; n = 3). All data are presented as the mean ± SD, $*p < 0.05$, $**p < 0.01$, $***p < 0.001$.

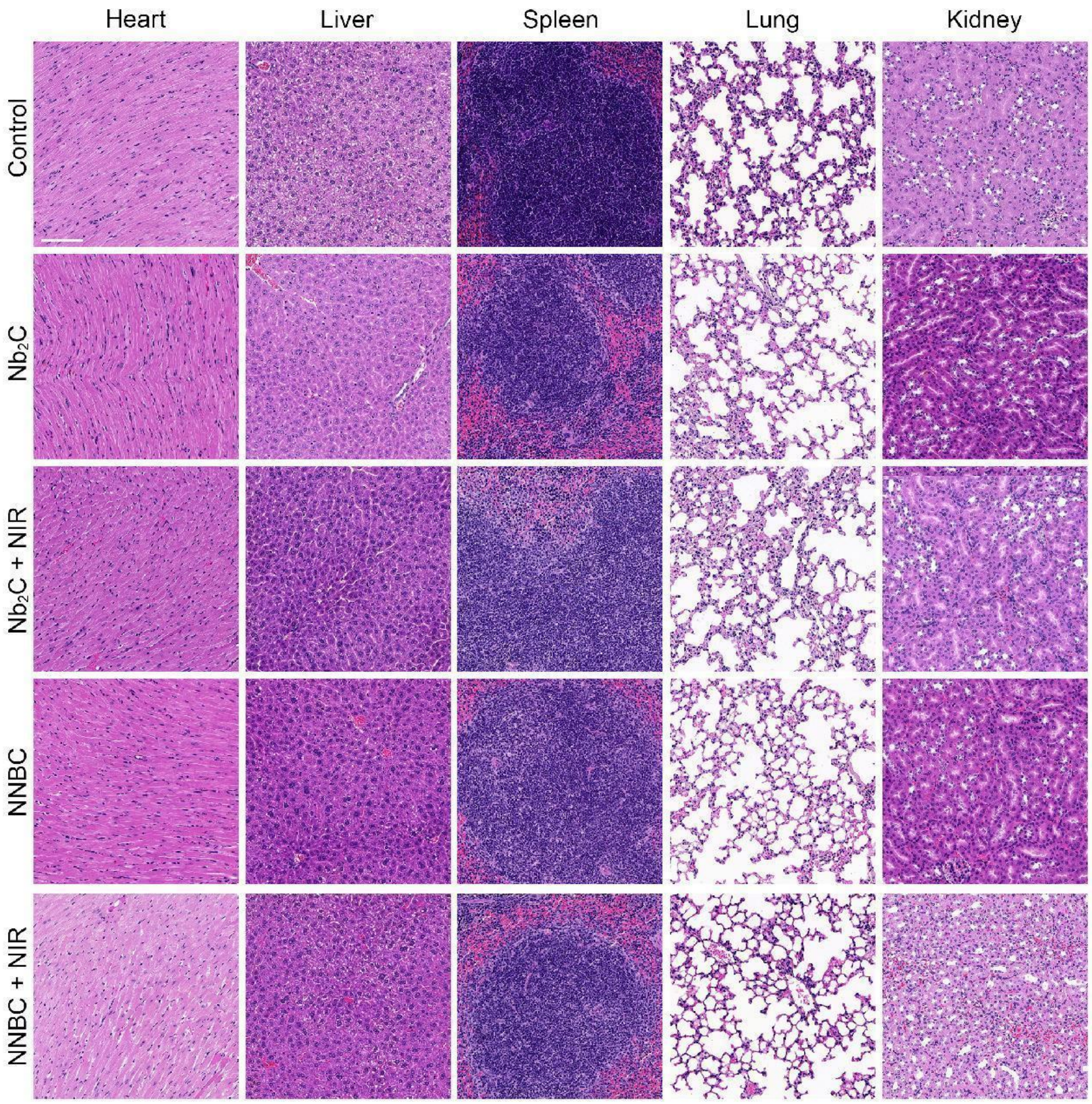

Figure S24. H&E staining images of major organs harvested from the skin wound models after 14 days' treatment (scale bar = 50 μm).

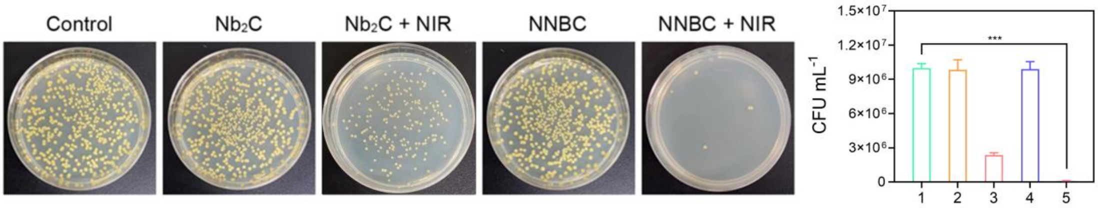

Figure S25. Photographs of *S. aureus* inoculated from bone tissues on agar plates and the corresponding bacterial activity of various treatments (1: Control; 2: Nb_2C; 3: Nb_2C + NIR; 4: NNBC; 5: NNBC + NIR; n = 3). All data are presented as the mean ± SD, *p < 0.05, **p < 0.01, ***p < 0.001.
